# Synergistic enhancement of electrocatalytic nitroarene hydrogenation over Mo_2_C@MoS_2_ heteronanorods with dual active-sites†

**DOI:** 10.1039/d3sc06010a

**Published:** 2024-02-06

**Authors:** Wanling Zhang, Wenbiao Zhang, Kun Yu, Jingwen Tan, Yi Tang, Qingsheng Gao

**Affiliations:** a College of Chemistry and Materials Science, Guangdong Provincial Key Laboratory of Functional Supramolecular Coordination Materials and Applications, Jinan University Guangzhou 510632 P. R. China wbzhang1994@hotmail.com tqsgao@jnu.edu.cn; b Department of Chemistry, Shanghai Key Laboratory of Molecular Catalysis and Innovative Materials, Laboratory of Advanced Materials and Collaborative Innovation Centre of Chemistry for Energy Materials, Fudan University Shanghai 200433 P. R. China

## Abstract

Electrocatalytic hydrogenation (ECH) enables the sustainable production of chemicals under ambient conditions, in which catalysts catering for the different chemisorption of reactants/intermediates are desired but still challenging. Here, Mo_2_C@MoS_2_ heteronanorods with dual active-sites are developed to accomplish efficient nitroarene ECH according to our theoretical prediction that the binding of atomic H and nitro substrates would be synergistically strengthened on Mo_2_C–MoS_2_ interfaces. They afford high faradaic efficiency (>85%), yield (>78%) and selectivity (>99%) for the reduction of 4-nitrostyrene (4-NS) to 4-vinylaniline (4-VA) in neutral electrolytes, outperforming not only the single-component counterparts of Mo_2_C nanorods and MoS_2_ nanosheets, but also recently reported noble-metals. Accordingly, *in situ* Raman spectroscopy combined with electrochemical tests clarifies the rapid ECH of 4-NS on Mo_2_C–MoS_2_ interfaces due to the facilitated elementary steps, quickly refreshing active sites for continuous electrocatalysis. Mo_2_C@MoS_2_ further confirms efficient and selective ECH toward functional anilines with other well-retained reducible groups in wide substrate scope, underscoring the promise of dual-site engineering for exploring catalysts.

## Abstract 2

Interfacial enhancement is evidenced on Mo_2_C@MoS_2_ heteronanorods for the efficient nitroarenes ECH, which promotes the bindings of H* and nitro substrates on Mo_2_C–MoS_2_ interfaces toward the fast elementary steps of the Langmuir–Hinshelwood mechanism.

## Graphical abstract

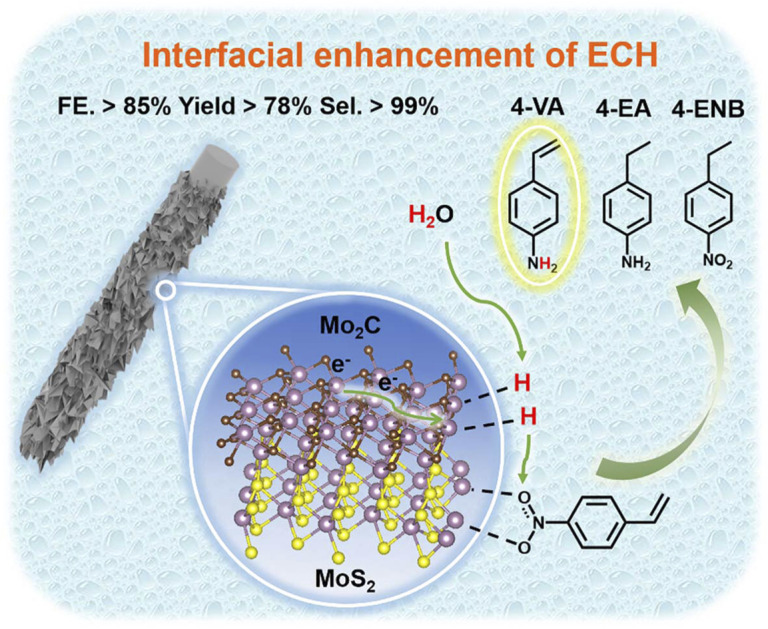

## Introduction

Renewable electricity powered chemical processes are burgeoning for the sustainable production of high-value chemicals.^1–3^ In particular, electrocatalytic hydrogenation (ECH) utilizes electrochemically formed hydrogen H* (* denotes an active site) from protons/water to upgrade various groups (*e.g.*, carbonyl, nitro, alkenyl, *etc.*) under ambient conditions,^4–9^ avoiding harsh operations associated with high temperature and H_2_ pressure adopted in thermocatalysis. For example, the reduction of nitroarenes to anilines is significant for manufacturing medicine, dyes, spices, and explosives.^10^ Witnessed progress has been made in the thermocatalytic hydrogenation of nitroarenes using H_2_,^11–13^ but it is challenged by the trade-off between activity and selectivity due to the difficult control over reaction pathways under heated and pressurized conditions.^10,14^ ECH, at room temperature and atmospheric pressure, neatly offers a controllable alternative to accommodate such multi-electron transfer processes *via* varying working potentials or currents.^6,15^ Following a typical Langmuir–Hinshelwood (L–H) mechanism,^16,17^ ECH proceeds *via* the stepwise reactions of adsorbed organics and H* on the catalyst surface, highly dependent on their competitive chemisorption. As H* is dominant, the hydrogen evolution reaction (HER) *via* its self-combination occurs easily, decreasing the faradaic efficiency (FE) of ECH,^18^ while the bimolecular coupling of nitroarenes or aldehydes/ketones toward azoxybenzenes or pinanols would exceed the target hydrogenation when the coverage of adsorbed organics is excessively high.^19,20^ It is necessary to design a catalyst surface that can counterbalance the difference in chemisorption toward efficient hydrogenation.^21,22^

As a noble-metal-free electrocatalyst, MoS_2_ demonstrates its efficiency in the ECH of aldehydes and nitroarenes;^16,23^ however, the sluggish H_2_O activation and discharging to H* (H_2_O + e^−^ →H* + OH^−^) under basic/neutral conditions unfortunately limit ECH kinetics. Efforts have been devoted to creating lattice defects and adjusting the phase transition,^23,24^ which fail to balance the competitive chemisorption of H* and organic substrates due to non-selective enhancement on a single kind of active sites. Constructing heterointerfaces involving dual active sites would be a promising alternative. The combination of MoS_2_ with noble-metals is capable of accommodating the different elementary steps of multi-electron transfer processes (*e.g.*, N_2_ and H_2_O reduction),^25–27^ but cannot meet the requirement of ECH because of the seriously retarded FEs and increased operation cost after introducing precious elements highly active for the HER.^28^

Thanks to the relatively more favorable H_2_O activation and stronger binding with H*,^29–31^ noble-metal-like molybdenum carbides (Mo_*x*_C) would be available to construct qualified interfaces on MoS_2_. Moreover, such two moieties share the same Mo element, enabling the facile construction and proliferative synergy of their interfaces. Here, Mo_2_C@MoS_2_ with dual sites was developed to enable the efficient ECH of nitroarenes. As indicated by density functional theory (DFT) calculations, the binding of H* and 4-nitrostyrene (4-NS*) was strengthened on the Mo_2_C and nearby MoS_2_ of Mo_2_C–MoS_2_ interfaces, respectively, which would promote the surface elementary steps of hydrogenation. As expected, Mo_2_C@MoS_2_ heteronanorods consisting of Mo_2_C axes and MoS_2_ shells accomplished high FE (>85%), selectivity (>99%) and yield (>78%) for the ECH of 4-NS to 4-vinylaniline (4-VA) in neutral electrolytes at −0.05 to −0.45 V *vs.* RHE, superior to single-component Mo_2_C nanorods and MoS_2_ nanosheets and even recently reported noble-metals. *In situ* Raman spectroscopy further confirmed the rapid ECH of 4-NS on Mo_2_C–MoS_2_ interfaces, quickly refreshing active sites for continuous electrocatalysis. In sharp comparison, the slow turnover due to the absence of either effective nitro activation on Mo_2_C or rich H* on MoS_2_ spawned the accumulation of hydrophobic reactants/intermediates and consequently an extra interfacial impedance on electrodes verified by electrochemical impedance spectroscopy (EIS). Moreover, Mo_2_C@MoS_2_ afforded efficient and selective ECH toward functional anilines in wide substrate scope, highlighting its efficiency for electrocatalytic refinery.

## Experimental section

### Chemicals

Ammonium molybdate tetrahydrate ((NH_4_)_6_Mo_7_O_24_·4H_2_O, AHM), aniline, thiourea and hydrochloric acid (HCl, AR) were purchased from Sinopharm Chemical Reagent Co. Ltd (Shanghai, China). 4-Nitrostyrene and Nafion solution (5 wt% in lower aliphatic alcohols and water) were purchased from Sigma-Aldrich. All chemicals were of analytical grade and used as received without further purification. All aqueous solutions were prepared using ultrapure water (>18.2 MΩ).

### Material synthesis

#### Synthesis of Mo_2_C nanorods

The synthesis of Mo_2_C nanorods was conducted according to our previous report.^32^ Specifically, 2.48 g AHM and 3.23 mL aniline were dissolved in 40 mL ultrapure water and adjusted to pH 4.0–4.5 by adding 1.0 mol L^−1^ HCl. The above solution was sealed and transferred to an oil bath at 50 °C for 5 hours while being stirred. Then, the as-received Mo_3_O_10_C_6_H_8_N_2_·2H_2_O nanowires were gathered and rinsed several times with ethanol and deionized water, respectively, and dried in a vacuum oven. To fabricate Mo_2_C nanorods, the above precursors (0.15 g) were annealed under an Ar flow at 775 °C for 5 hours.

#### Synthesis of MoS_2_ nanosheets

AHM (0.35 g) and thiourea (1.83 g) were dissolved in 10 mL of ultrapure water in a 20-mL Teflon-lined stainless-steel autoclave and heated at 240 °C for 3 hours. Afterwards, the solid was washed and collected by centrifugation with both water and ethanol several times. MoS_2_ nanosheets were finally obtained by overnight drying at 50 °C under vacuum.

#### Synthesis of Mo_2_C@MoS_2_ nanorods

Mo_2_C@MoS_2_ nanorods were synthesized *via* a controllable surface oxidation of Mo_2_C nanorods followed by a hydrothermal sulfidation approach. In detail, the above prepared Mo_2_C nanorods (0.3 g) were placed in a crucible and oxidized at 200 °C under an air flow for 30 min. And then, the obtained samples (0.1 g) and thiourea (1.0 g) were dispersed and ultrasonicated in 10 mL of water to obtain the homogeneous solution. Subsequently, this solution was transferred into a 20 mL Teflon-lined stainless-steel autoclave and heated at 240 °C for 3 hours. Finally, the black solid was washed and collected by centrifugation with water and ethanol several times. Mo_2_C@MoS_2_ nanorods were finally received after drying at 50 °C in a vacuum overnight.

### Physical characterization

X-ray diffraction (XRD) analysis was conducted on a Bruker D8 Advance diffractometer with Cu Kα radiation (*λ* = 1.54056 Å). Transmission electron microscopy (TEM) and scanning electron microscopy (SEM) were conducted on a JEOL JEM-2100F and ZEISS ULTRA 55, respectively. X-ray photoelectron spectroscopy (XPS) was carried out on a Thermo Fisher Scientific (Escalab 250Xi), and the binding energy was calibrated with respect to the C 1s (284.6 eV) of contaminated carbon. Raman spectra were collected on a LabRAM HR Evolution, with an excitation laser wavelength of 532 nm. Nitrogen adsorption–desorption isotherms were used to determine the specific surface areas of electrocatalysts on an automatic gas adsorption analyzer (Quantachrome Autosorb-iQ-MP) by a multipoint Brunauer–Emmett–Teller (BET) method. Electron paramagnetic resonance (EPR) spectra were recorded on a Magnettech ESR500.

### Electrochemical test

To prepare working electrodes, 10 mg of electrocatalysts and 100 μL of 5 wt% Nafion solution were dispersed in 1.0 mL water–ethanol with a *V*_water_/*V*_ethanol_ ratio of 4 : 1 and then the mixture was vigorously sonicated for at least 30 min to get a homogeneous ink. The as-obtained ink (500 μL) was loaded onto pre-treated carbon cloth (1.5 cm × 1.5 cm) carefully and dried naturally in air. The working electrode was directly subjected to the ECH of nitroarenes, which was conducted on a CHI 650E electrochemical workstation (Chenhua Instruments Shanghai Co., Ltd, China), within a gas-tight two-compartment electrochemical cell equipped with a piece of a proton-exchange membrane (Nafion 117) as the separator. A saturated calomel electrode (SCE) and a platinum electrode were used as the reference and counter electrodes, respectively. The cathodic electrolyte (pH 6.8) contained 4-NS (12.5 mmol L^−1^), LiClO_4_ (0.1 mol L^−1^) and methanol/water (1 : 1), which was degassed using a N_2_ gas flow for 30 min prior to the ECH and saturated with the same flow-rate N_2_ during the test. Methanol was used to improve the homogeneous dispersion of the 4-NS phase in the electrolyte. Linear sweep voltammetry (LSV) was conducted in an undivided cell with and without 12.5 mmol L^−1^ 4-NS in 40 mL LiClO_4_ solutions (pH 6.8).

To identify the involvement of H* during the ECH, EPR measurement with 5,5-dimethyl-1-pyrroline-N-oxide (DMPO) as the trapping agent was conducted. After the chronoamperometry test at a given constant potential of −0.45 V *vs.* RHE for 5 min, 0.1 mM DMPO was added into the cathodic electrolyte and stirred for 1 min. Afterwards, the electrolyte was taken out for the EPR test. Meanwhile, *t*-BuOH (25 wt% in 0.1 M LiClO_4_) was used to quench hydrogen radicals during chronoamperometry at −0.45 V *vs.* RHE, in which 4-VA was detected every 60 min. The obviously prohibited yield of 4-VA in comparison with that free-from *t*-BuOH again proved the involvement of H*.

### Product analysis

After reactions, liquid products were collected from the cathode compartment and analyzed *via* a HPLC (SHIMADZU LC-20AT) equipped with a UV detector at 254 and 370 nm. The column (INERTSUSTAIN C18) was operated at 40 °C and the binary gradient method was adopted at a flow rate of 0.8 mL min^−1^, which contained H_2_O and CH_3_OH (*V*_CH_3_OH_ : *V*_H_2_O_ = 6 : 4). The FE (%), yield (%) and selectivity (%) of products were calculated based on the following equations:
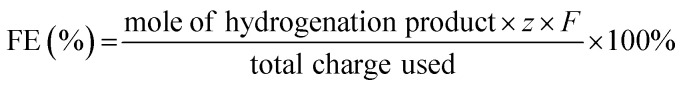

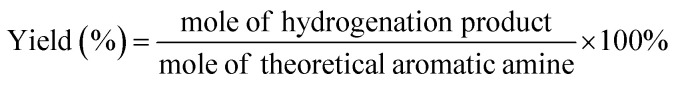

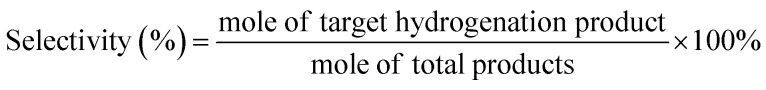
where, *z* is the number of transferred electrons, which for 4-VA, 4-ethyl-nitrobenzene (4-ENB) and 4-ethylaniline (4-EA) is 6, 2 and 8, respectively. *F* is the faradaic constant (96 485 C mol^−1^).

### Theoretical calculations

In this work, all the theoretical computations were based on the DFT method, as implemented in the CASTEP package of Materials Studio. For the exchange–correlation functional, we used the generalized gradient approximation (GGA) with the Perdew–Burke–Ernzerhof (PBE) functional. The projector augmented wave (PAW) method was applied to depict the interaction between ion and electron, and the cut of energy was set at 400.0 eV. The force and energy cutoff were set at 0.01 eV Å^−1^ and 10^−6^ eV for the geometry optimization, respectively. A 5 × 5 × 1 Monkhorst–Pack grid *k*-point sampling was conducted for the bulk and slab structure optimization. The optimized bulk Mo_2_C (space group: *P*6_3_/*mmc*; *a* = *b* = 3.0 Å, *c* = 4.7 Å) and 2H–MoS_2_ (space group: *P*6_3_/*mmc*; *a* = *b* = 3.2 Å, *c* = 12.3 Å) were selected. Then, we selected a 3 × 3 relaxed rhombus MoS_2_(002) bilayer on top of the relaxed 4 × 4 Mo_2_C(001) surface to model Mo_2_C–MoS_2_ heterojunctions, in which S atoms were introduced to saturate the coordination of Mo atoms on the Mo_2_C(001). The adsorption slab would be curved as the (010) of the Mo_2_C–MoS_2_ layer and a vacuum layer of 12 Å was used to isolate the slab as the boundary condition, which made the interactions between neighboring slabs negligible according to other previous work.^33^

The binding energy (BE) of an adsorbate was calculated as:BE_(adsorbate)_ = *E*_(slab+adsorbate)_ − *E*_(slab)_ − *E*_(adsorbate)_where *E*_(slab+adsorbate)_, *E*_(slab)_ and *E*_(adsorbate)_ are the energies of a slab with the adsorbate and the pure slab/facet and the adsorbate in the gas phase, respectively. The *E*(H) is taken as one half the total energy of the H_2_ molecule during the calculation of H binding energy.

## Results and discussion

**Fig. 1:**
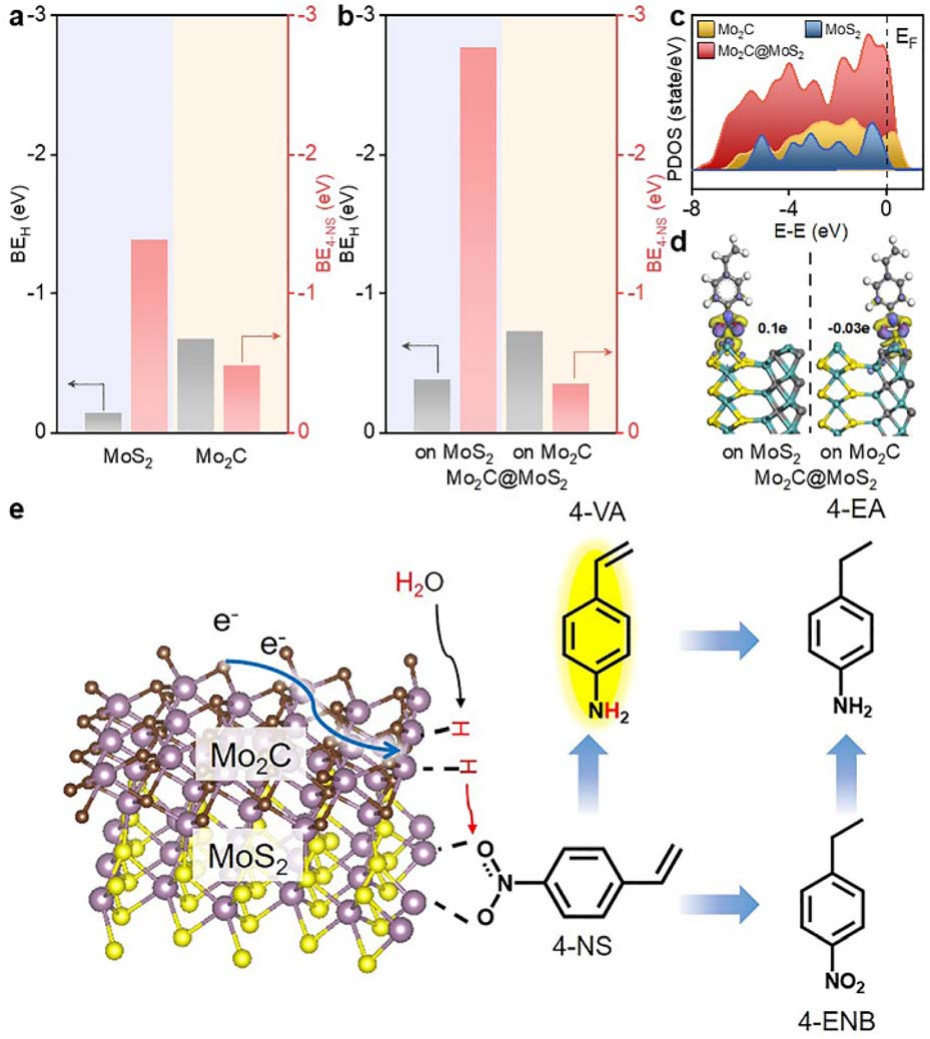
BE_H_ and BE_4-NS_ on (a) Mo_2_C and MoS_2_, and (b) Mo_2_C–MoS_2_ interfaces. (c) PDOS of Mo_2_C, Mo_2_C–MoS_2_ and MoS_2_. (d) Electron density difference of Mo_2_C@MoS_2_ with 4-NS adsorbed on the Mo_2_C or MoS_2_ moiety. (e) Schematic illustration for the boosted ECH of 4-NS on Mo_2_C–MoS_2_ interfaces.

DFT calculations were conducted to analyze the varied binding energies of H* (BE_H_) and 4-NS* (BE_4-NS_) on MoS_2_, Mo_2_C and their interfaces. According to previous reports^34,35^ and the experimentally evidenced selective hydrogenation of the nitro group in this work, the vertical chemisorption of 4-NS *via* bonding nitro with Mo sites was reasonably taken into account.^36^ In further regard to H*, the Mo atom on the Mo_2_C(101) surface was indicated as the available site because of the obviously stronger BE_H_ (−0.68 eV) than that on a carbon site (+0.19 eV) (Fig. S1, ESI†), which also exceeded those on the Mo (−0.14 eV) and S sites (−0.1 eV) of MoS_2_. For a reliable comparison, the Mo sites on Mo_2_C, MoS_2_ and Mo_2_C–MoS_2_ models were further considered in the context of the competitive chemisorption of 4-NS* and H*. As depicted in Fig. 1a, the edge-site rich MoS_2_(010) presented obviously weaker binding with H* (−0.14 eV) but the stronger one with 4-NS* (−1.39 eV) relative to Mo_2_C (101) (BE_H_: −0.68 eV; BE_4-NS_: −0.48 eV), which suggested the favorable activation of nitro on MoS_2_ and the rich H* on Mo_2_C when these sites were combined together. As expected, a Mo_2_C–MoS_2_ interface showed the synchronously strengthened BE_H_ (−0.73 eV) on Mo_2_C and BE_4-NS_ (−2.77 eV) on MoS_2_ (Fig. 1b), in favor of the ECH *via* the L-H mechanism. Accordingly, the projected density of states (PDOS) for d bands (Fig. 1c) was visibly broadened near the Fermi level (*E*_F_) on Mo_2_C–MoS_2_, benefiting the chemisorption of either H* or 4-NS* due to the easy overlap of orbitals. In the chemisorption configurations (Fig. 1d), the electron density distribution of 4-NS* on MoS_2_ was closer than that on Mo_2_C, confirming its more favorable activation on the MoS_2_ moiety. It's thus reasonable to design Mo_2_C–MoS_2_ interfaces to boost 4-NS reduction thanks to the capability of counterbalancing the difference between H* and 4-NS* (Fig. 1e). The ECH of 4-NS can produce 4-VA and 4-ENB *via* selectively hydrogenating nitro and vinyl groups, respectively, both of which would be further reduced to 4-EA. 4-VA is targeted due to its high added-value for chemical synthesis.

**Fig. 2:**
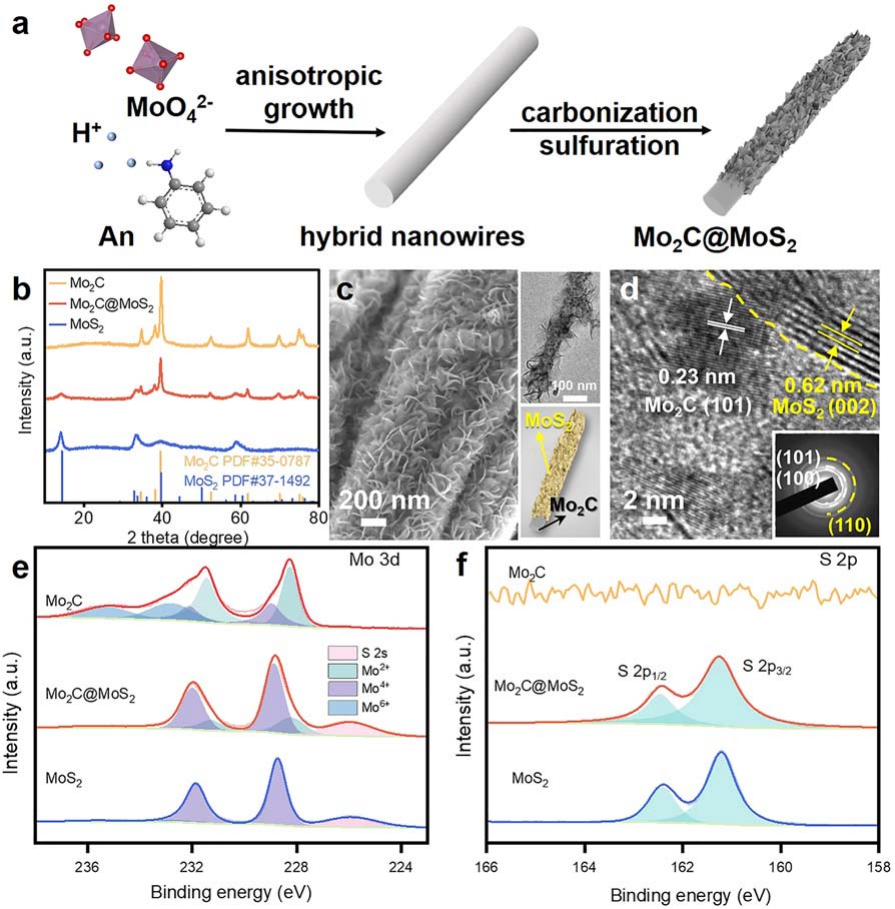
(a) Schematic illustration for the preparation of hierarchical Mo_2_C@MoS_2_ nanorods. (b)XRD patterns, (c) SEM and (d) TEM images of Mo_2_C@MoS_2_ heteronanorods. (e) Mo 3d, (f) S 2p XPS spectra of Mo_2_C, Mo_2_C@MoS_2_ and MoS_2_.

Here, the sequent carbonization and sulfidation of Mo_3_O_10_(C_6_H_8_N)_2_·2H_2_O nanowires (Fig. S2, ESI†) was employed to fabricate Mo_2_C@MoS_2_ heteronanorods for efficient hydrogenation (Fig. 2a). XRD analysis confirmed the co-presence of Mo_2_C and MoS_2_ phases in Mo_2_C@MoS_2_ (Fig. 2b), in which the (100), (002) and (101) of Mo_2_C and the (002), (101) and (110) of MoS_2_ were observed. Accordingly, it showed the characteristic E^1^_2g_, A_1g_ and 2LA(M) vibration modes of MoS_2_, while Mo_2_C was transparent in Raman spectra (Fig. S3, ESI†). As shown in SEM and TEM (Fig. 2c), Mo_2_C@MoS_2_ retained the rod-like morphology of parent Mo_2_C, but generated nanosheet shells on the surface, resulting in a hierarchical structure that combines the structural merits of 1D Mo_2_C (Fig. S4, ESI†) and 2D MoS_2_ (Fig. S5, ESI†). High-resolution TEM (HR-TEM) clearly identified the Mo_2_C–MoS_2_ interfaces with visible Mo_2_C(101) and MoS_2_(002) lattices (Fig. 2d). Besides, the corresponding elemental mapping confirmed the uniform distribution of Mo, S, and C in Mo_2_C@MoS_2_ (Fig. S6, ESI†). Such a hierarchical 1D@2D nanostructure enabled its relatively larger surface area of 39.5 m^2^ g^−1^ (Fig. S7, ESI†), as compared to Mo_2_C (24.9 m^2^ g^−1^) and MoS_2_ (25.0 m^2^ g^−1^). Moreover, a series of Mo_2_C@MoS_2_ prepared with different feeding ratios had a similar nanostructure (Fig. S8 and S9, ESI†). In addition, the chemical states of elements were analyzed by XPS (Fig. 2e, f and Table S1, ESI†). In the deconvoluted Mo 3d profile, the doublets at 228.3 and 231.4 eV were ascribed to the Mo 3d_5/2_ and Mo 3d_3/2_ of Mo^2+^ due to the presence of Mo_2_C, and those at 228.7 and 231.8 eV corresponded to Mo^4+^ of MoS_2_.^37^ The S 2p profile also indicated the consistent chemical state of S in Mo_2_C@MoS_2_ and MoS_2_.

**Fig. 3:**
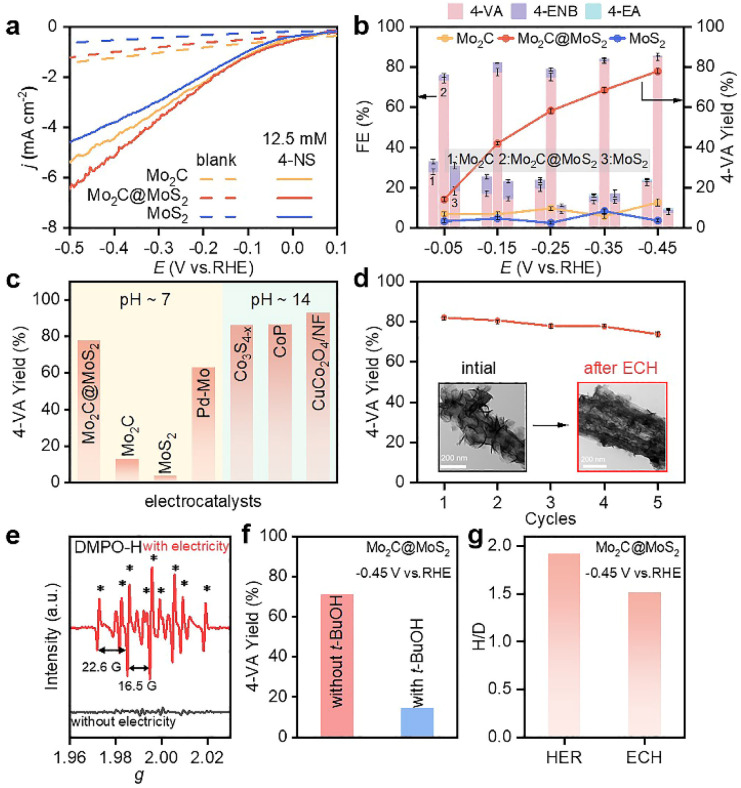
(a) Polarization curves of Mo_2_C, Mo_2_C@MoS_2_ and MoS_2_ in 0.1 M LiClO_4_ with and without 12.5 mM 4-NS, and (b) product FEs and yields at −0.05 to −0.45 V *vs.* RHE with 12.5 mM 4-NS (reaction time: 5 h). (c) Comparison of 4-VA yield of Mo_2_C@MoS_2_ with that of state-of-the-art electrocatalysts, including Pd–Mo,^39^ Co_3_S_4−*x*_,^35^ CoP,^40^ and CuCo_2_O_4_/NF.^41^ (d) Cycle-dependent 4-VA yield over Mo_2_C@MoS_2_ at −0.45 V. The insets of panel d show the TEM images of Mo_2_C@MoS_2_ before and after the test. (e) Quasi *in situ* EPR trapping of hydrogen radicals over Mo_2_C@MoS_2_. (f) Yield of 4-VA with and without *t*-BuOH as the hydrogen radical scavenger. (g) Kinetic isotopic effect of Mo_2_C@MoS_2_ for the HER and ECH.

The ECH performances of Mo_2_C, MoS_2_ and Mo_2_C@MoS_2_ were tested in a H-type cell separated by a proton exchange membrane (Nafion 117). Cyclic voltammetry (CV) curves were collected in 0.1 M LiClO_4_ solution (pH = 6.8) containing 50 wt% methanol to improve the solubility of 4-NS (Fig. S10, ESI†), which exhibited a cathodic reduction peak at −0.29 to −0.50 V *vs.* RHE, corresponding to the stepwise reduction.^38^ Then, their polarization curves in 0.1 M LiClO_4_ with and without 4-NS were compared. The cathodic currents of all three catalysts increased after introducing 4-NS and that of Mo_2_C@MoS_2_ was the highest (Fig. 3a), indicative of its outstanding activity for the ECH. Subsequently, chronoamperometry was performed at −0.05 to −0.45 V *vs.* RHE for 5 h (Fig. 3b). Mo_2_C@MoS_2_ maintained an obviously higher FE and yield of 4-VA, as compared to Mo_2_C and MoS_2_. The total FEs were lower than 100% associated with the concomitant HER. At −0.45 V *vs.* RHE, the FE, yield and selectivity of 4-VA were as high as ∼85%, ∼80%, and 99%, respectively (Fig. 3b and S11, ESI†). Furthermore, the electrochemical surface areas (ECSAs) of these electrodes were visualized through calculating the double-layer capacitances (*C*_dl_) according to the proportional relationship (Fig. S12, ESI†) and subsequently used for normalizing the rate of 4-VA production to access the difference in specific activity. The higher specific reaction rate on Mo_2_C@MoS_2_ than those on Mo_2_C and MoS_2_ indicated the intrinsic superiority associated with Mo_2_C–MoS_2_ interfaces (Fig. S13, ESI†). In further comparison with recently reported electrocatalysts including noble-metals (Fig. 3c and Table S2, ESI†), Mo_2_C@MoS_2_ performed among the best and featured mild operation with neutral electrolytes, avoiding the use of alkalis that easily trigger by-reactions (*e.g.*, bi-molecular coupling to azoxybenzenes) and equipment corrosion.

The time-dependent conversion of 4-NS to 4-VA on Mo_2_C@MoS_2_ was in accordance with the visible decrease of 4-NS and the quick emergence of 4-VA in the HPLC chromatogram (Fig. S14–S16, ESI†). The by-product of 4-ENB, derived from the hydrogenation of the vinyl group, was limited to a low level, confirming the highly selective hydrogenation of the nitro group due to the vertical chemisorption. Moreover, Mo_2_C@MoS_2_ showed stability in repeated ECH tests (Fig. 3d). The slight decrease of 4-VA yield in each cycle should be ascribed to the inevitable loss of the catalyst in the processes of cleaning. The post-test characterization studies (*e.g.*, TEM, XRD and XPS) identified the negligible change of both hierarchical nanostructures and surface states (insets of Fig. 3d, S17 and S18, ESI†). In addition, the series of Mo_2_C@MoS_2_ obtained with the varied feeding ratio showed enhanced activity with the increased amount of thiourea (Fig. S19, ESI†), probably thanks to the enriched interfaces.

In order to detect the active species of ECH, quasi *in situ* electron paramagnetic resonance (EPR) spectroscopy coupled with electrochemical tests was conducted with 5,5-dimethyl-1-pyrroline-N-oxide (DMPO) as the trapping agent (Fig. 3e). The characteristic EPR signals of DMPO-H adducts (nine peaks, *α*_N_ = 16.5 G and *α*_H_ = 22.6 G) were visible at −0.45 V *vs.* RHE, suggesting that the active H* species was electrochemically generated *via* water reduction.^39^ Accordingly, when *tert*-butanol (*t*-BuOH) was introduced as a hydrogen radical scavenger, the conversion of 4-NS decreased significantly (Fig. 3f and S20, ESI†), confirming H* as the key intermediate.^42^ Furthermore, the kinetic isotopic effect (KIE) was tested for both the HER and ECH on Mo_2_C@MoS_2_, in terms of the ratio of current densities within H_2_O and D_2_O at −0.45 V *vs.* RHE (Fig. S21, ESI†). Mo_2_C@MoS_2_ showed a value of 1.92 for the HER, higher than that for ECH (1.52) (Fig. 3g). It's suggested that the H* is not the only determinant in the ECH. Meanwhile, the productivity of 4-VA on Mo_2_C@MoS_2_ presented a positively logarithmical correlation with the initial 4-NS concentration (Fig. S22, ESI†), and the CVs of 4-NS showed a good linear correlation of anodic peak currents with scan rates (Fig. S23, ESI†), which together confirmed the nonnegligible contribution of 4-NS*.^16,43^ The strengthened 4-NS* adsorption on Mo_2_C@MoS_2_ was evidenced by the more significant increase in open-circuit potential (73.1 mV) after injecting 4-NS into the blank electrolyte (Fig. S24, ESI†), as compared with those observed on Mo_2_C (0.3 mV) and MoS_2_ (26.5 mV). This experimental result consistent with our theoretical prediction (Fig. 1b) indicated the effective chemisorption and activation of the nitro group. According to the typical L–H mechanism, the conversion of 4-NS to 4-VA requires the co-participation of both 4-NS* and H*, which thereby highly depends on the competitive adsorption of these two substrates. A strong 4-NS* adsorption will inevitably weaken one of the H* and *vice versa*. As verified by the above experimental and theoretical results, the Mo_2_C–MoS_2_ interface is conducive to excellent catalytic performance because of its capability of balancing the competitive adsorption of H* and 4-NS*.

**Fig. 4:**
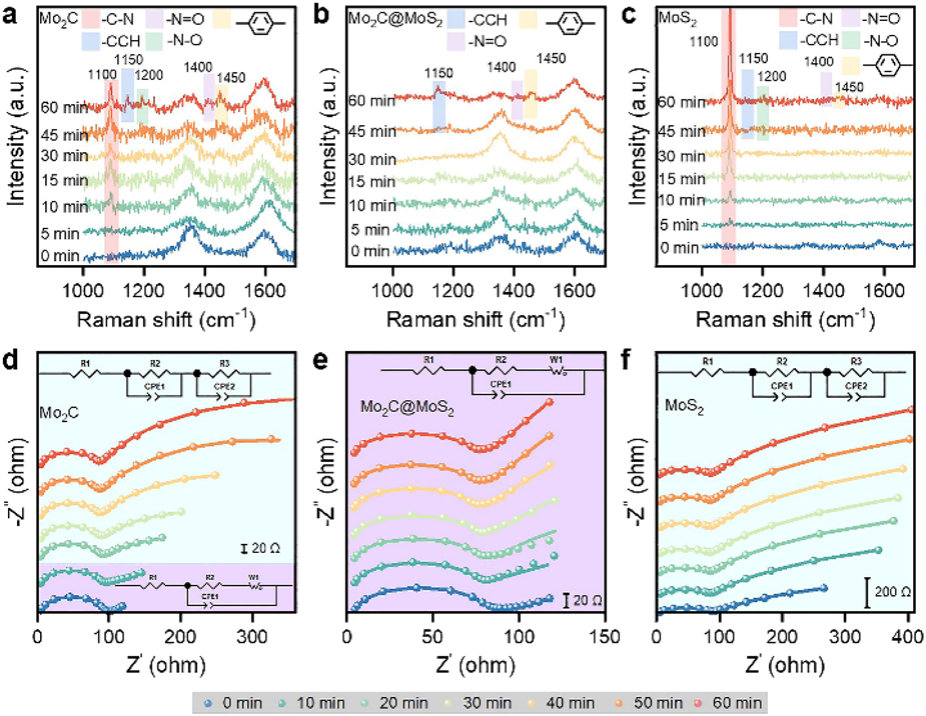
(a–c) *In situ* Raman spectra and (d–f) Nyquist plots collected on (a and d) Mo_2_C, (b and e) Mo_2_C@MoS_2_, and (c and f) MoS_2_ at −0.45 V *vs.* RHE.

We collected *in situ* Raman spectra on Mo_2_C, Mo_2_C@MoS_2_ and MoS_2_ to understand their varied ECH performance (Fig. 4a–c). The initial spectra at 0 min exhibited two major signals at about 1350 and 1600 cm^−1^ attributing to the carbon matrix of Mo_2_C and Mo_2_C@MoS_2_ (Fig. S25, ESI†). The ECH of 4-NS occurred as reaction time went on, and accordingly new bands appearing at 1100, 1200, 1400, 1450, and 1150 cm^−1^ could be attributed to the stretching vibrations of C–N, N–O, N=O and benzene ring and the in-plane bending one of CCH, respectively (Table S3, ESI†).^44,45^ In detail, the band of C–N (1100 cm^−1^) was detected after 5–10 min on Mo_2_C and MoS_2_, and its progressive enhancement on MoS_2_ suggested the accumulation of 4-NS due to the strong chemisorption of 4-NS* but insufficient *H for the ECH. Owing to the relatively weaker binding with 4-NS* on Mo_2_C, the C–N band increased slowly. Besides, the others assigned to CCH, N–O, N=O and benzene ring were due to the presence of intermediates, such as phenylhydroxylamine and nitroso benzene. By contrast, there were nearly no signals of 4-NS and intermediates detected on Mo_2_C@MoS_2_ before 60 min, confirming the rapid surface elementary steps of ECH involving the co-presented H* and 4-NS* on Mo_2_C–MoS_2_ interfaces.

Quasi *in situ* EIS, an efficient technique for identifying reaction interfaces, was performed at intervals during the ECH (Fig. 4d, e and S26, ESI†). The series resistance (*R*_s_) was consistent over Mo_2_C, Mo_2_C@MoS_2_ and MoS_2_ (Table S4, ESI†), but the configurations at low frequencies were quite different, which were well-fitted with the varied equivalent circuit diagram. It's shown that the kinetic behavior of Mo_2_C has changed from infinite diffusion to multiple interface diffusion after 20 min and MoS_2_ kept the feature of multiple interface diffusion. In sharp comparison, Mo_2_C@MoS_2_ showed kinetic behavior approximating infinite diffusion, consistent with the result of *in situ* Raman spectroscopy. In other words, the consistent interfacial charge transfer on Mo_2_C@MoS_2_ is ascribed to the quickly refreshed active surface; however, those of Mo_2_C and MoS_2_ suffer from an extra interfacial impedance owing to reactant/intermediate accumulation on their surfaces.

We further examined the efficiency of Mo_2_C@MoS_2_ in wide substrate scope (Table 1) and analyzed the products by ^1^H NMR (Fig. S27–S46, ESI†). For *meta* (*m*) and *para* (*p*) -halogenated nitrobenzenes (X = I, Br, Cl), Mo_2_C@MoS_2_ exhibited excellent performance with high FE (84–99%) and considerable yield (72–96%) of target halogenated anilines, in which the by-products of hydro-dehalogenation and H_2_ were rarely detected. This demonstrated the extraordinary tolerance for halogen functional groups. Moreover, it achieved the excellent efficacy of ECH for nitroarenes with a methoxy group, keeping the high FE (∼99%) and yield (88–92%) of target anilines. These results verified the promise of Mo_2_C@MoS_2_ for the ECH of nitroarenes.

**Table 1:** ECH of various functionalized nitroarenes over Mo_2_C@MoS_2_

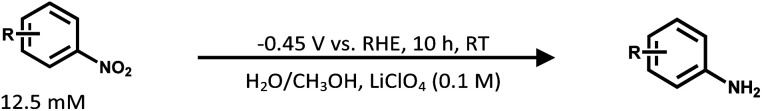		
R=Cl	FE 90% yield 88% (*m*)	FE 87% yield 72% (*p*)
R=Br	FE 84% yield 80% (*m*)	FE 86% yield 82% (*p*)
R=I	FE 99% yield 96% (*m*)	FE 99% yield 84% (*p*)
R=OCH_3_	FE 99% yield 92% (*m*)	FE 99% yield 88% (*p*)

## Conclusions

In summary, Mo_2_C@MoS_2_ heteronanorods with dual sites were proposed to enable the efficient ECH of nitroarenes on synergized interfaces. As predicated using DFT calculations, the Mo_2_C–MoS_2_ interface can strengthen the binding of H* on Mo_2_C and 4-NS* on nearby MoS_2_, conducive for the successive hydrogenation toward the corresponding anilines. Mo_2_C@MoS_2_ achieved the high FE (>85%), yield (>78%) and selectivity (>99%) of 4-NS to value-added 4-VA, superior to Mo_2_C and MoS_2_. Accordingly, *in situ* Raman spectroscopy and quasi *in situ* EIS confirmed the facilitated chemisorption and further hydrogenation of 4-NS on Mo_2_C–MoS_2_ interfaces owing to the synergistically strengthened binding. Moreover, Mo_2_C@MoS_2_ delivered high efficiency for the ECH of nitroarenes to produce functional anilines in wide substrate scope. This work will provide new opportunities to develop high-performance electrocatalysts *via* the rational engineering of nanostructures and interfaces. Nevertheless, there still existed some challenges for ECH in comparison with thermo-catalysis, such as relatively lower space-time yield and immature reactor design. Drawing lessons from the mature experience of thermal-catalysis will expedite further progress in ECH.

## Data availability

Data supporting the findings of this study are available within the article ESI.†

## Author contributions

Wanling Zhang: synthesis, investigations, formal analysis, writing of the original draft, data curation. Wenbiao Zhang: writing-review and editing, DFT calculations, supervision. Kun Yu: experimental analysis, image polish. Jingwen Tan: proofreading of the original draft. Yi Tang: conceptualization, supervision, funding acquisition. Qingsheng Gao: conceptualization, supervision, writing-review and editing, funding acquisition.

## Conflicts of interest

There are no conflicts to declare.

## Supplementary Material

SC-015-D3SC06010A-s001
