# Supplementary material for: Synergistic enhancement of electrocatalytic nitroarene hydrogenation over Mo2C@MoS2 heteronanorods with dual active-sites
Source: Chem Sci. 2024 Feb 6;15(10):3446–52. doi: 10.1039/d3sc06010a (PMC10915856; doi:10.1039/d3sc06010a)
Supplement: SC-015-D3SC06010A-s001 [file SC-015-D3SC06010A-s001.pdf]

Electronic Supplementary Material (ESI) for Chemical Science.
This journal is © The Royal Society of Chemistry 2024

Electronic Supplementary Information

Synergistic Enhancement of Electrocatalytic Nitroarenes Hydrogenation over $Mo_2C@MoS_2$ Heteronanorods with Dual Active-sites

Wanling Zhang,[a] Wenbiao Zhang,*[a,b] Kun Yu,[a] Jingwen Tan,[a] Yi Tang,[b] and Qingsheng Gao*[a]

[a] College of Chemistry and Materials Science, Guangdong Provincial Key Laboratory of Functional Supramolecular Coordination Materials and Applications, Jinan University, Guangzhou 510632, P. R. China. E-mail: wbzhang1994@hotmail.com; tqsgao@jnu.edu.cn

[b] Department of Chemistry, Shanghai Key Laboratory of Molecular Catalysis and Innovative Materials, Laboratory of Advanced Materials and Collaborative Innovation Center of Chemistry for Energy Materials, Fudan University, Shanghai 200433, China.

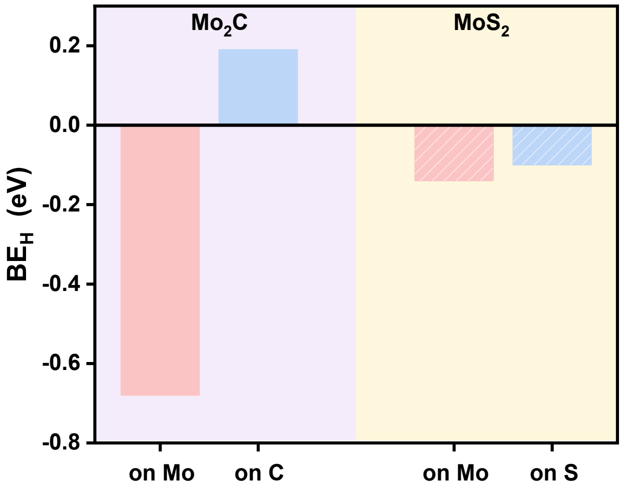

Fig. S1. BE_H on Mo_2C(101) and MoS_2(010) surface.

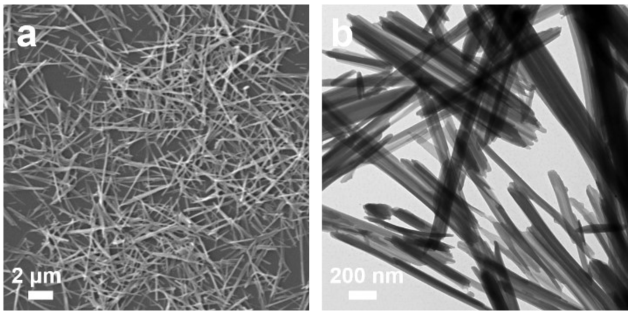

Fig. S2. (a) SEM and (B) TEM images of $Mo_3O_{10}(C_6H_8N)_2$•$2H_2O$ precursors.

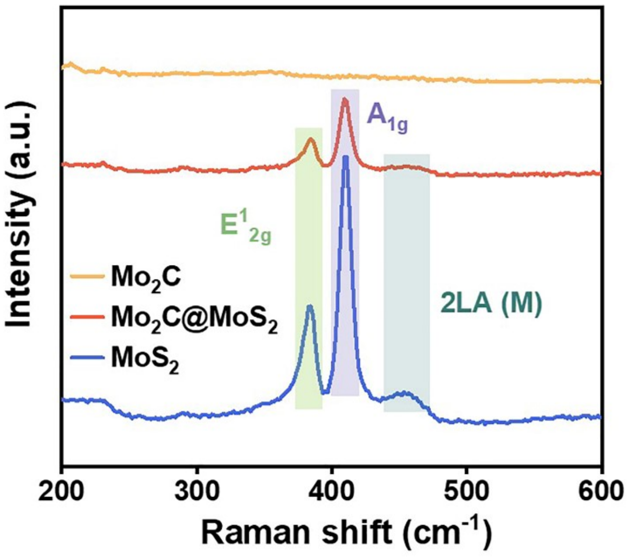

Fig. S3. Raman spectra of Mo_2C, $Mo_2C@MoS_2$ and MoS_2.

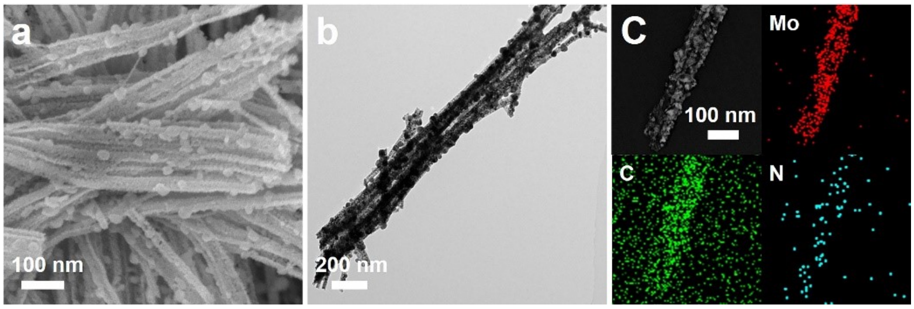

Fig. S4. (a) SEM and (b) TEM images, and (c) corresponding elemental mapping of Mo_2C nanorods.

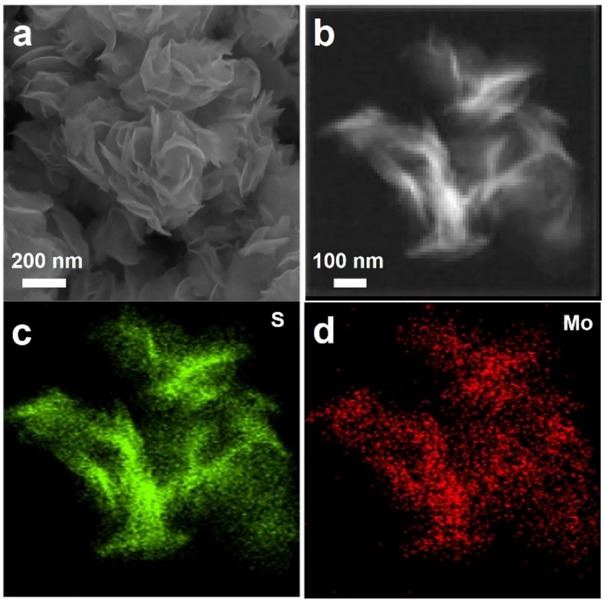

Fig. S5. (a) SEM and (b) TEM images, and (c-d) corresponding elemental mapping of MoS_2 nanosheets.

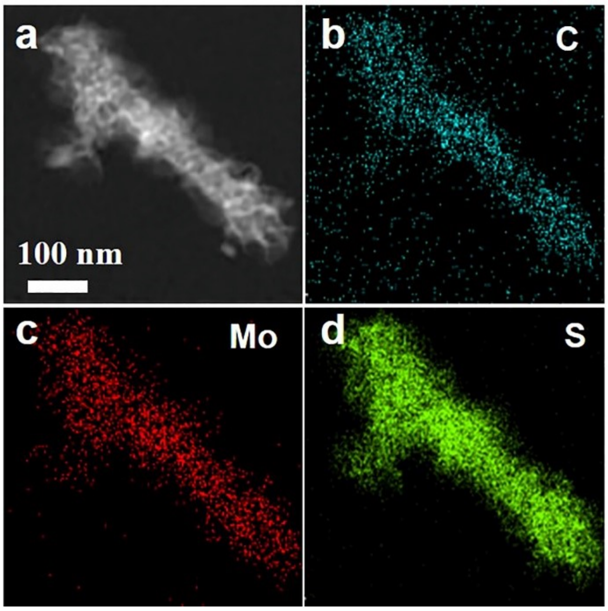

Fig. S6. Elemental mapping of $Mo_2C@MoS_2$ heteronanorods.

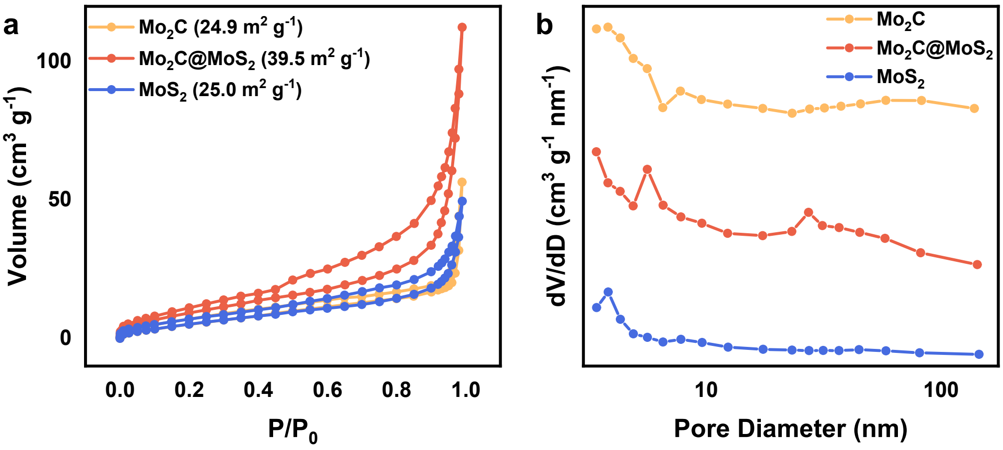

Fig. S7. (a) N_2 adsorption-desorption isotherms and (b) pore-size distribution derived from BJH model of Mo_2C, $Mo_2C@MoS_2$ and MoS_2. To avoid the false signals in pore-size distribution, the adsorption data, rather than desorption ones, was adopted for the calculation with the classical BJH model. As a result, the distribution of nanoporosity is not obvious, probably due to the random formation of pores by stacking 1D and 2D building blocks.

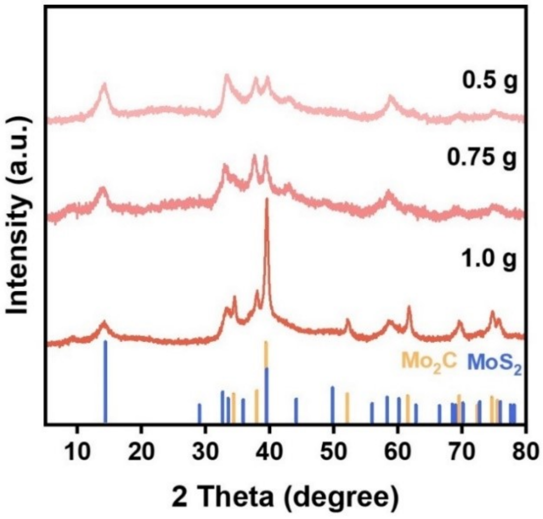

Fig. S8. XRD patterns of $Mo_2C@MoS_2$ fabricated with the varied feeding of thiourea (0.5 g, 0.75 g and 1.0 g).

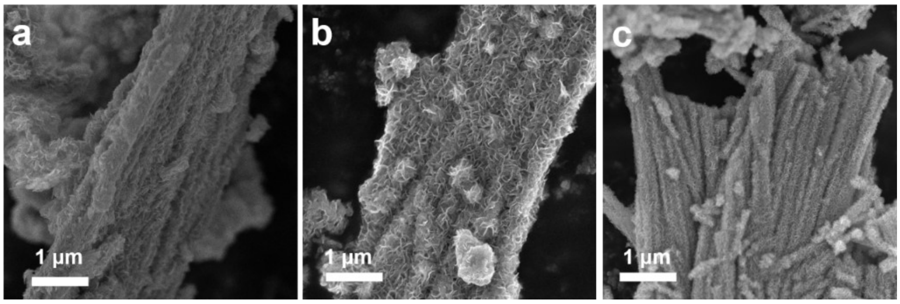

Fig. S9. SEM images of $Mo_2C@MoS_2$ fabricated with the varied feeding of thiourea: (a) 0.5 g, (b) 0.75 g and (c) 1.0 g.

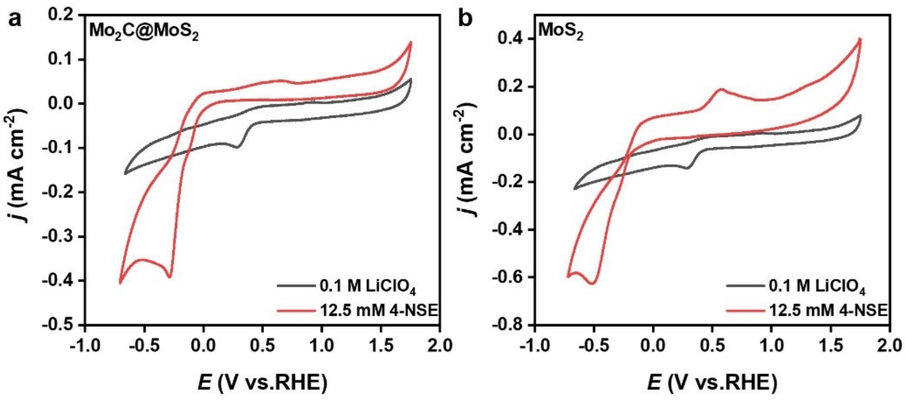

Fig. S10. CV curves of (a) $Mo_2C@MoS_2$ and (b) MoS_2 without or with adding 4-NS.

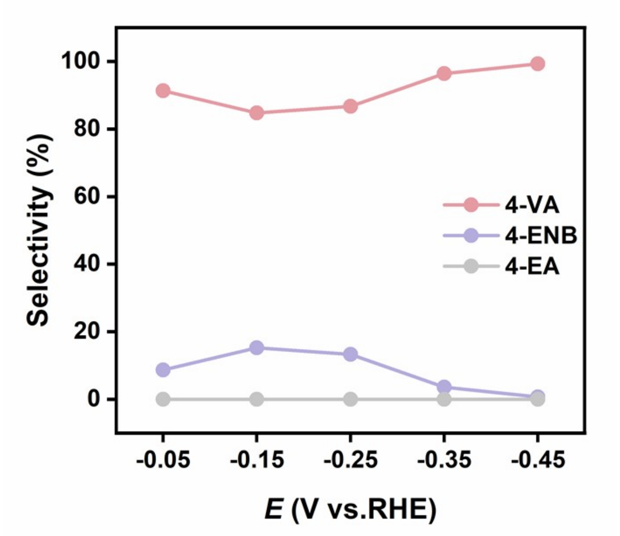

Fig. S11. Selectivity of $Mo_2C@MoS_2$ for ECH at -0.05 ~ -0.45 V vs. RHE in 0.1 M $LiClO_4$ with 12.5 mM 4-NS.

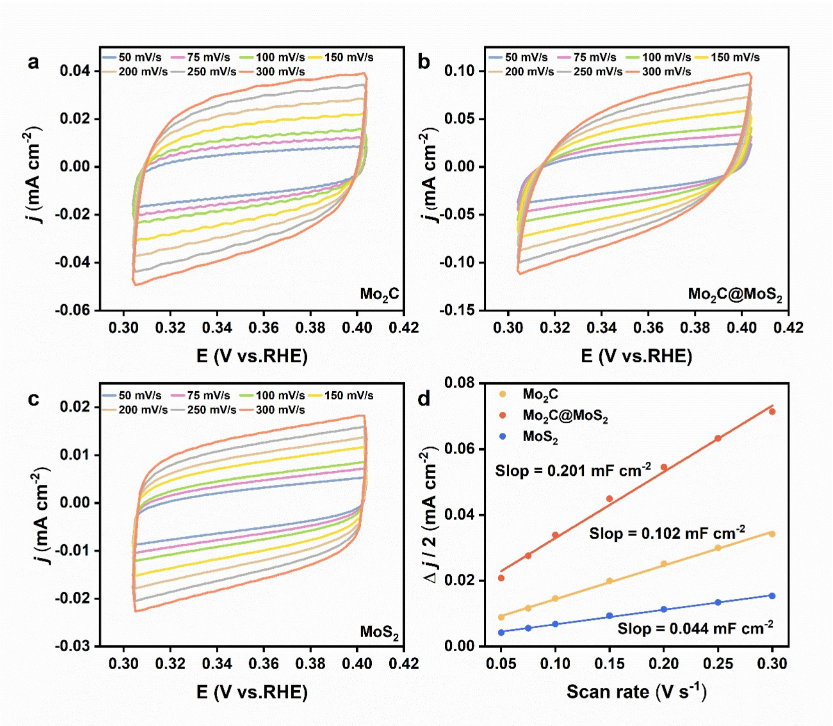

Fig. S12. CVs of (a) Mo_2C, (b) $Mo_2C@MoS_2$, and (c) MoS_2 in 0.1 M $LiClO_4$. (d) Estimation of C_{dl} of Mo_2C, $Mo_2C@MoS_2$ and MoS_2. Usually, the specific capacitance for a flat surface (1 cm^2) is in a range of 20-60 μF cm_{ECSA}^{-2}, and herein a moderate value of 40 μF cm_{ECSA}^{-2} is adopted to calculate ECSA and make comparison. The ECSA values of Mo_2C, $Mo_2C@MoS_2$ and MoS_2 were 5.738, 11.306 and 2.475

cm_{ECSA}^2, respectively.

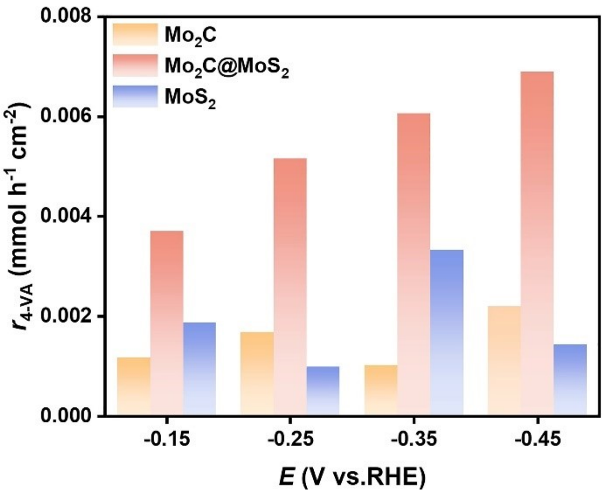

Fig. S13. Specific reaction rate of 4-VA production on Mo_2C, $Mo_2C@MoS_2$ and MoS_2 at -0.15 ~ 0.45 V vs. RHE. The specific rates were calculated by normalizing the productivity of 4-VA by the ECSA vales and reaction time.

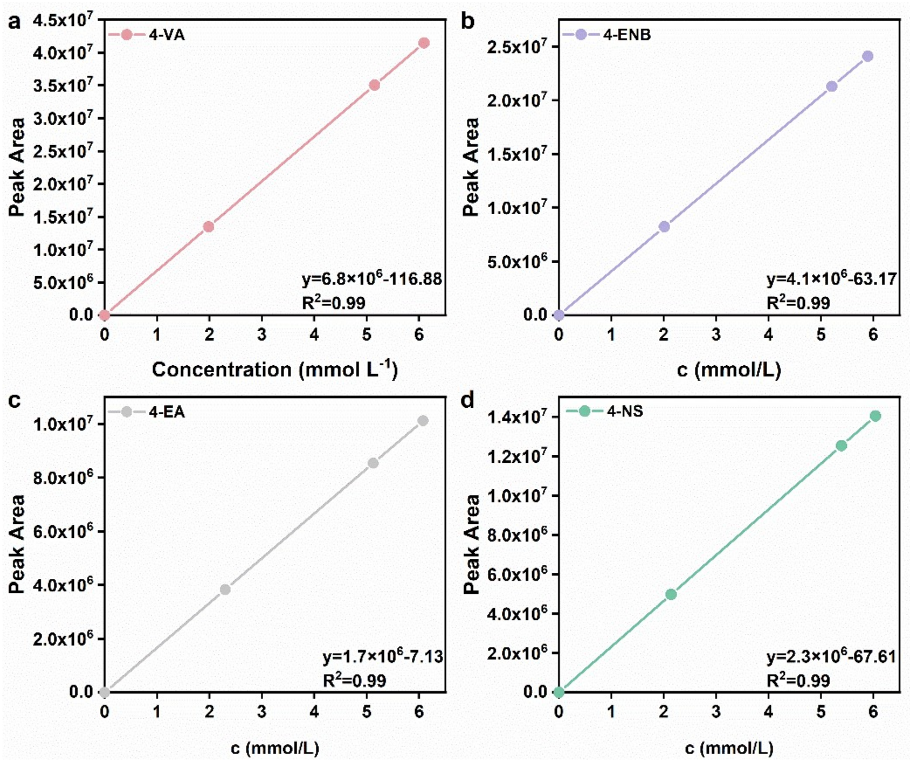

Fig. S14. HPLC standard curve of (a) 4-NS, (b) 4-VA, (c) 4-ENB and (d) 4-EA.

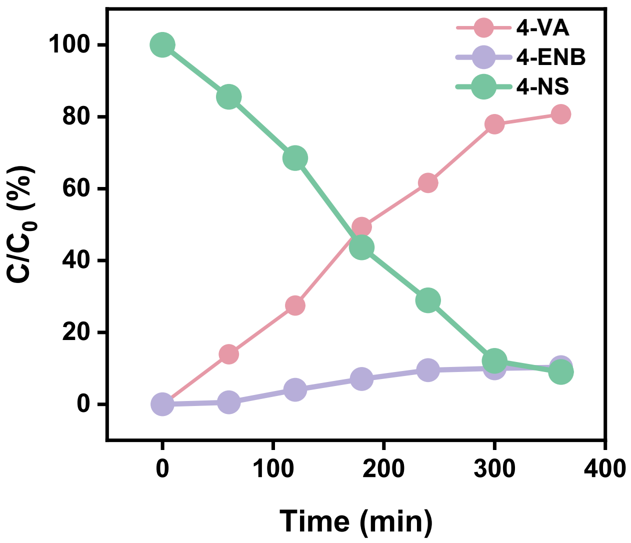

Fig. S15. Time-dependent conversion plots for the ECH of 4-NS into 4-VA and 4-ENB at -0.45 V vs. RHE over $Mo_2C@MoS_2$.

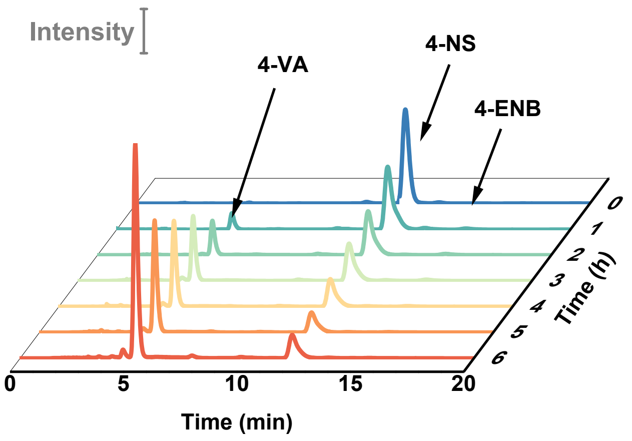

Fig. S16. HPLC chromatograms acquired at various electrolytic times during the ECH of 4-NS over $Mo_2C@MoS_2$ at -0.45V vs. RHE.

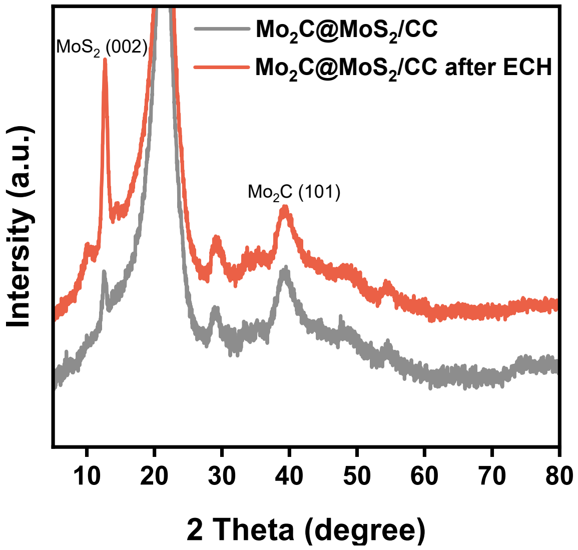

Fig. S17. XRD patterns of $Mo_2C@MoS_2$ before and after the ECH test.

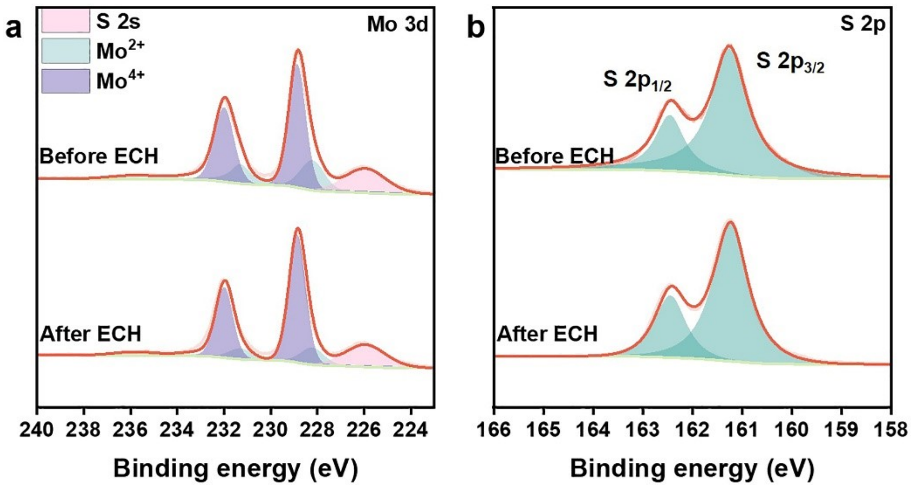

Fig. S18. (a) Mo 3d and (b) S 2p XPS profiles of $Mo_2C@MoS_2$ before and after the ECH test.

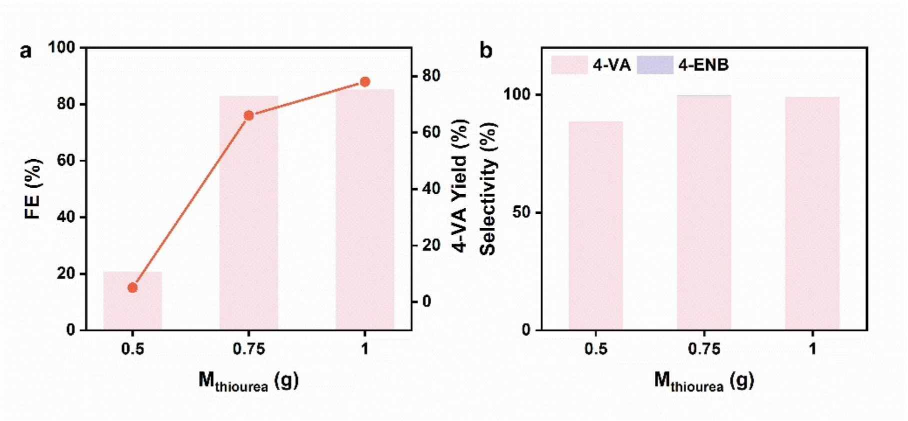

Fig. S19. (a) FEs and 4-VA yield and (b) selectivity of 4-NS ECH over various $Mo_2C@MoS_2$ synthesized with the varied thiourea feeding.

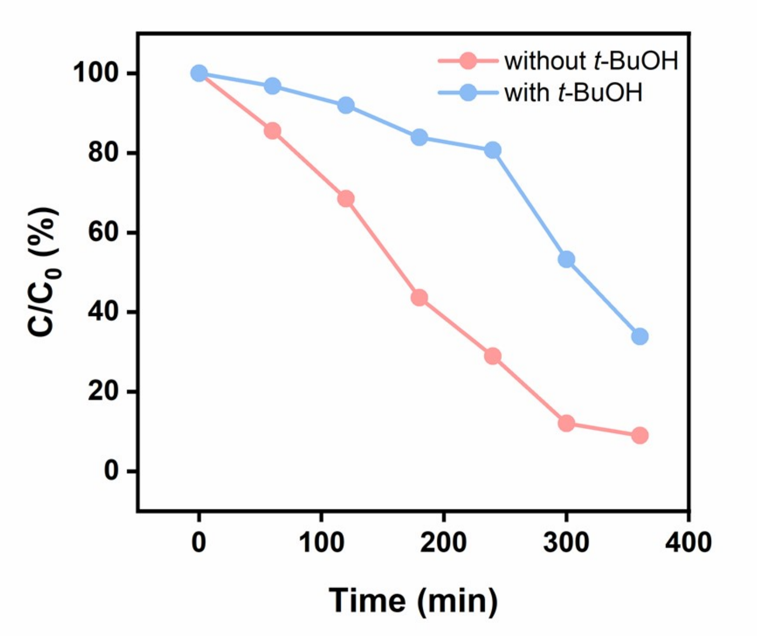

Fig. S20. Conversion of 4-NS over $Mo_2C@MoS_2$ at -0.45 V vs. RHE with and without the addition of *t*-BuOH.

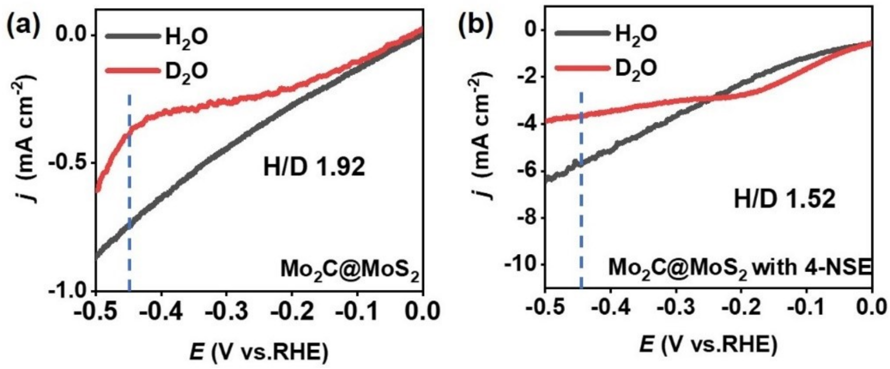

Fig. S21. Comparisom of LSV curves on $Mo_2C@MoS_2$ in H_2O and D_2O with and without the addition of 12.5 mM 4-NS at -0.45 V vs. RHE.

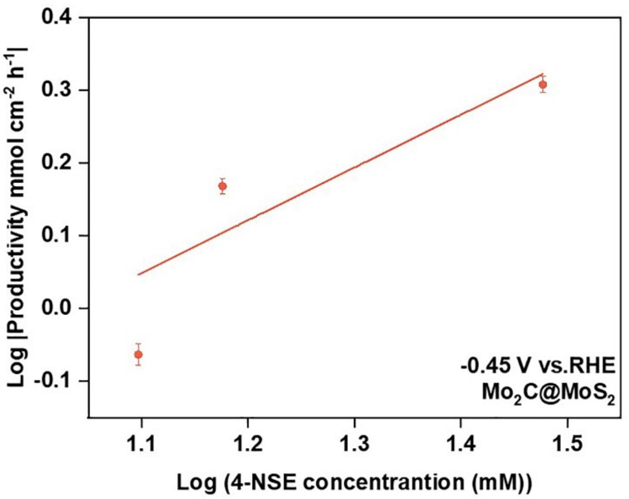

Fig. S22. Dependence of 4-VA yield on initial 4-NS concentration over $Mo_2C@MoS_2$.

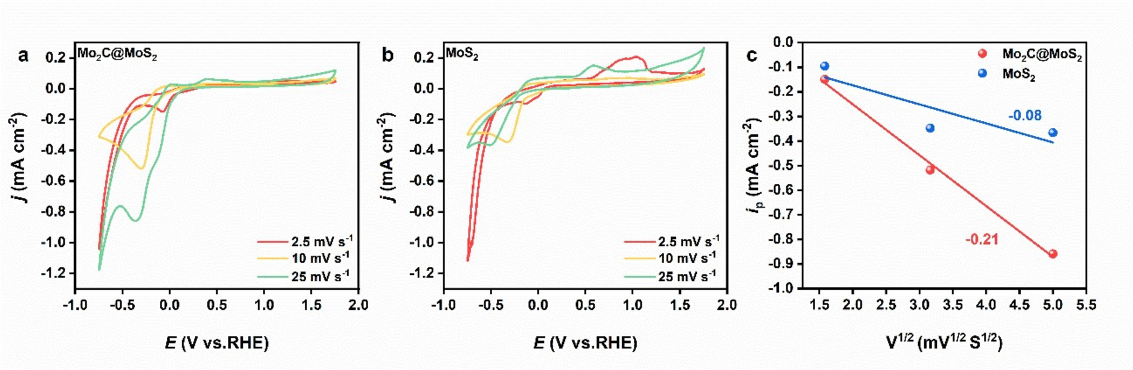

Fig. S23. CVs of (a) $Mo_2C@MoS_2$ and (b) MoS_2 with scan rates from 2.5 to 25 mV s^{-1}. (c) Plotting the reduction peak current density of 4-NS against the scan rate of CV measurements.

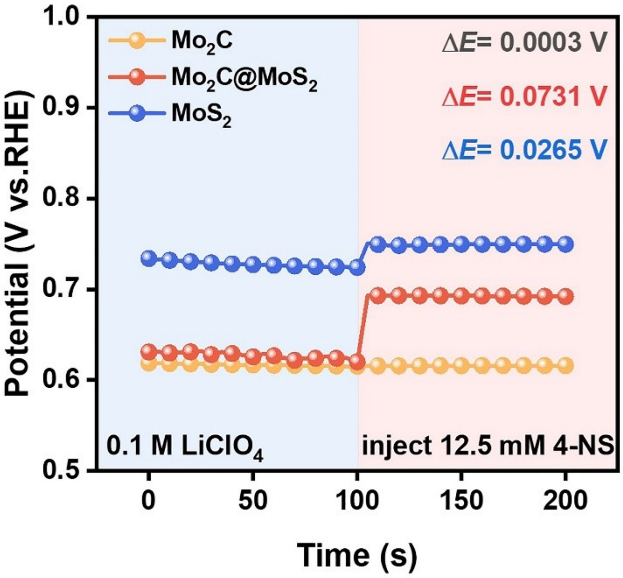

Fig. S24. OCP curves of Mo_2C, $Mo_2C@MoS_2$ and MoS_2 in 0.1 M $LiClO_4$ before and after injecting 12.5 mM 4-NS.

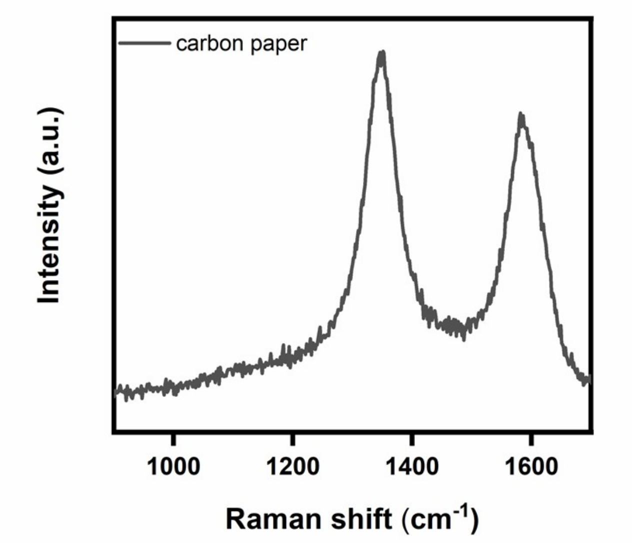

Fig. S25. The initial Raman spectra of $Mo_2C@MoS_2$ collected at 0 min in the ECH with 4-NS.

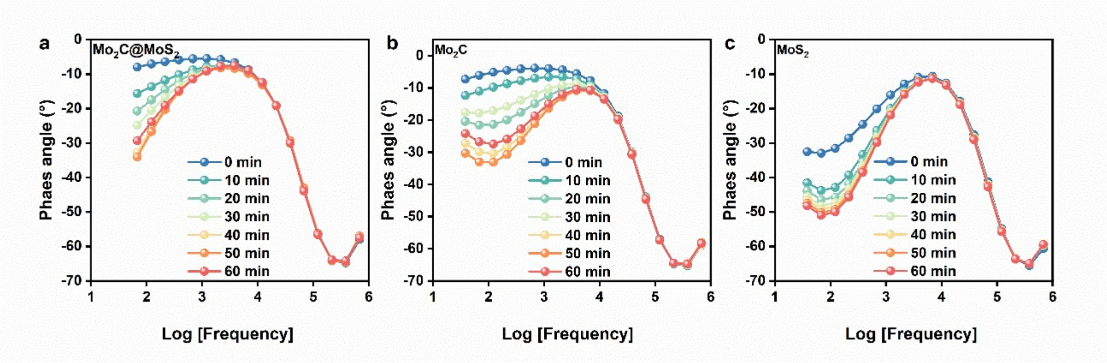

Fig. S26. Bode plots of (a) Mo_2C, (b) $Mo_2C@MoS_2$ and (c) MoS_2 collected at intervals during the ECH in 0.1 M $LiClO_4$ with 4-NS (-0.45 V vs. RHE).

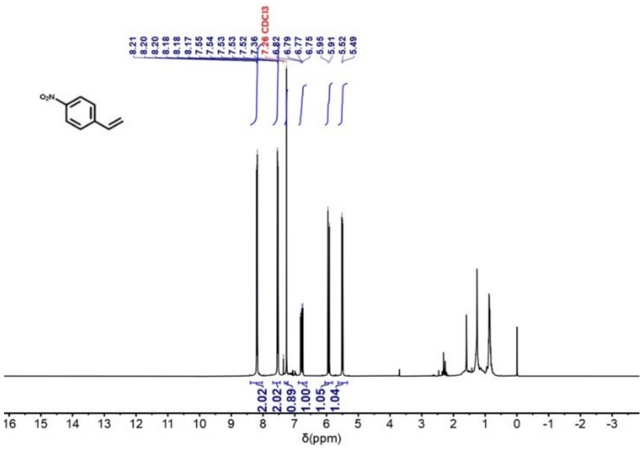

Fig. S27. ^{1}H NMR of 4-nitrostyrene. ^{1}H NMR (400 MHz, Chloroform-d) δ 8.40 – 8.03 (m, 2H), 7.69-7.46 (m, 2H), 6.78 (dd, *J* = 17.6, 10.9 Hz, 1H), 5.93 (d, *J* = 17.6 Hz, 1H), 5.50 (d, *J* = 10.9 Hz, 1H).

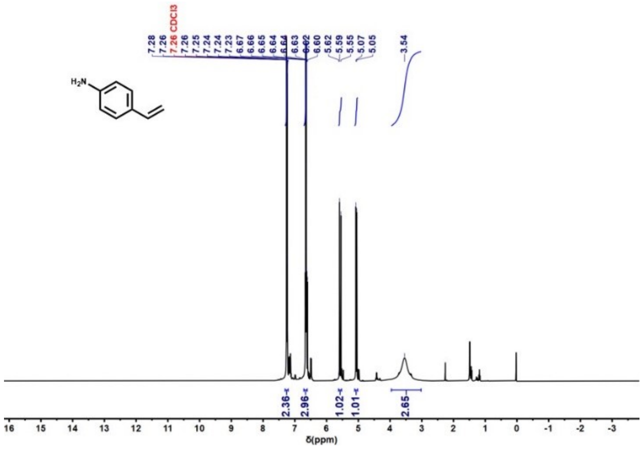

Fig. S28. ^{1}H NMR of 4-Vinylaniline. ^{1}H NMR (400 MHz, Chloroform-*d*) δ 7.31 – 7.21 (m, 2H), 6.72 – 6.61 (m, 3H), 5.57 (d, *J* = 17.6 Hz, 1H), 5.06 (d, *J* = 10.9 Hz, 1H), 3.54 (s, 3H).

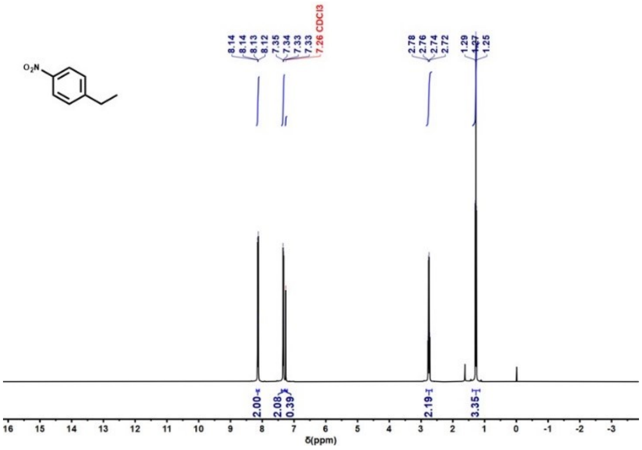

Fig. S29. ^{1}H NMR of 4-ethylnitrobenzene. ^{1}H NMR (400 MHz, Chloroform-*d*) δ 8.19 – 8.09 (m, 2H), 7.40 – 7.29 (m, 2H), 2.75 (q, *J* = 7.6 Hz, 2H), 1.27 (t, *J* = 7.6 Hz, 3H).

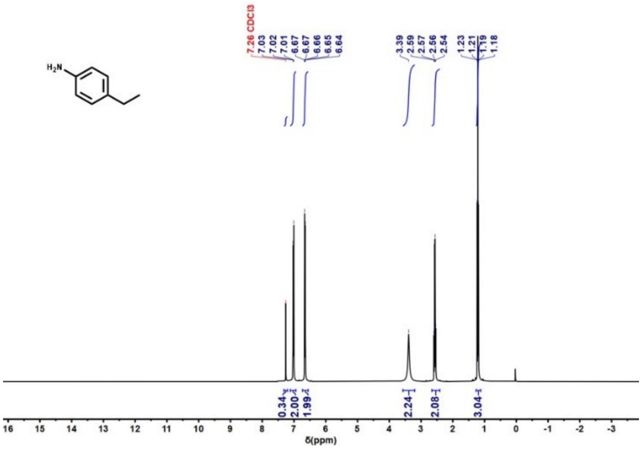

Fig. S30. ^{1}H NMR of 4-ethylaniline. ^{1}H NMR (400 MHz, Chloroform-d) δ 7.02 (d, J = 8.2 Hz, 2H), 6.73 – 6.55 (m, 2H), 3.39 (s, 2H), 2.57 (q, J = 7.6 Hz, 2H), 1.21 (t, J = 7.6 Hz, 3H).

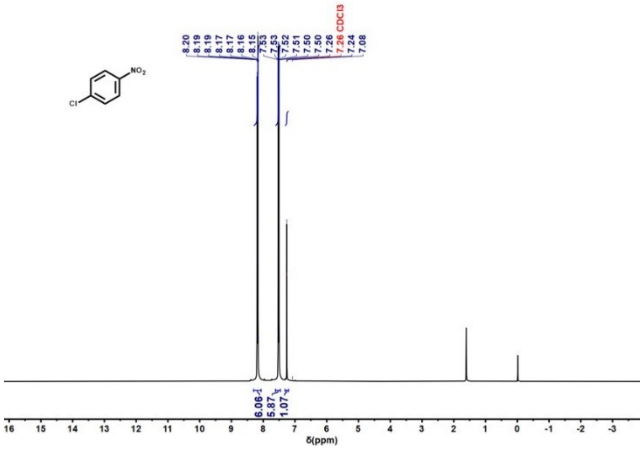

Fig. S31. ^{1}H NMR of P-Chloronitrobenzene. ^{1}H NMR (400 MHz, Chloroform-*d*) δ 8.30 – 8.07 (m, 6H), 7.61 – 7.46 (m, 6H), 7.26 (s, 1H).

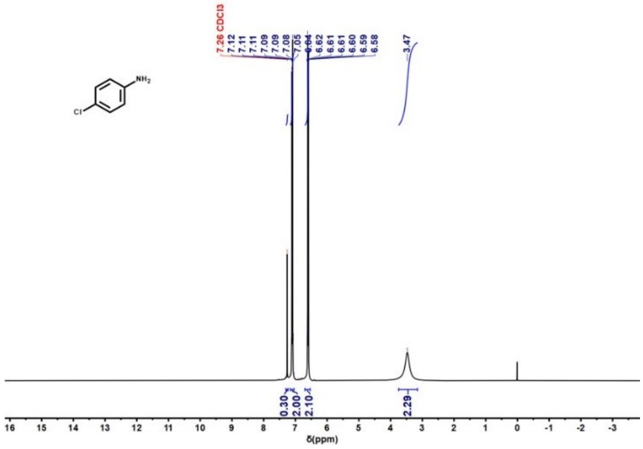

Fig. S32. ^{1}H NMR of p-Chloroaniline. ^{1}H NMR (400 MHz, Chloroform-*d*) δ 7.16 – 7.05 (m, 2H), 6.70 – 6.53 (m, 2H), 3.47 (s, 2H).

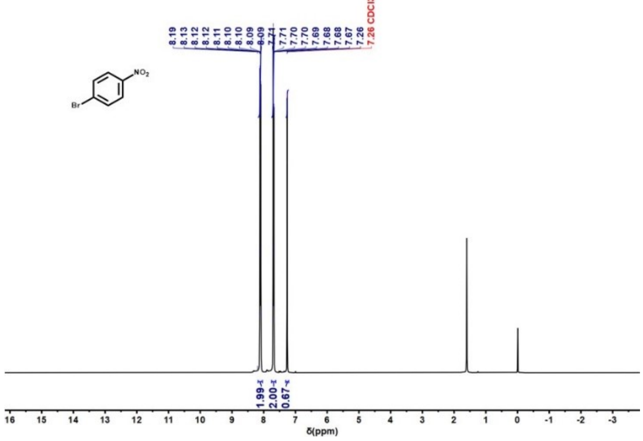

Fig. S33. ^{1}H NMR of P-Bromonitrobenzene. ^{1}H NMR (400 MHz, Chloroform-d) δ 8.18 – 8.04 (m, 2H), 7.75 – 7.62 (m, 2H), 7.26 (s, 1H).

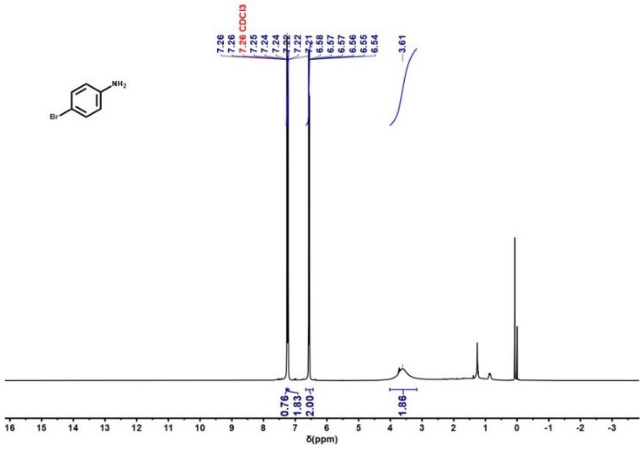

Fig. S34. ^{1}H NMR of P-bromoaniline. ^{1}H NMR (400 MHz, Chloroform-*d*) δ 7.26 (d, *J* = 1.0 Hz, 1H), 7.25 – 7.20 (m, 2H), 6.67 – 6.43 (m, 2H), 3.61 (s, 2H).

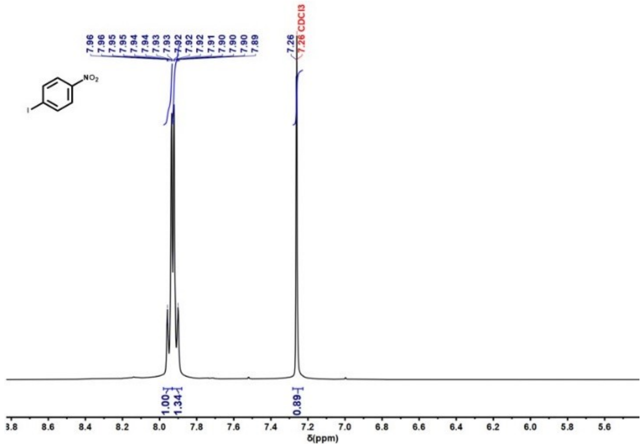

Fig. S35. ^{1}H NMR of P-iodonitrobenzene. ^{1}H NMR (400 MHz, Chloroform-*d*) δ 7.95 (dd, *J* = 9.3, 1.2 Hz, 1H), 7.91 (dd, *J* = 9.3, 1.2 Hz, 1H), 7.26 (s, 1H).

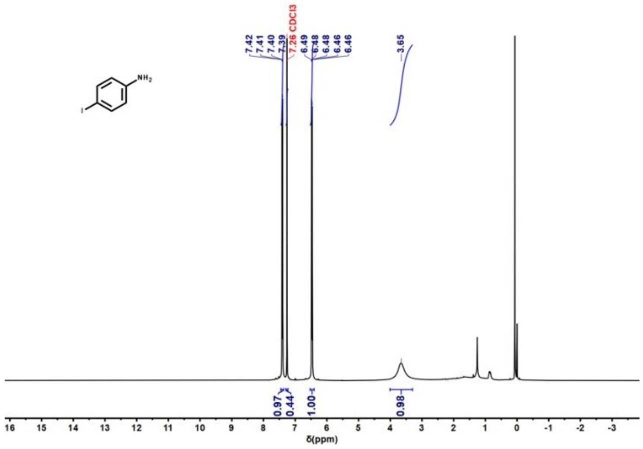

Fig. S36. ^{1}H NMR of p-Iodoaniline. ^{1}H NMR (400 MHz, Chloroform-*d*) δ 7.45 – 7.36 (m, 1H), 6.53 – 6.40 (m, 1H), 3.65 (s, 1H).

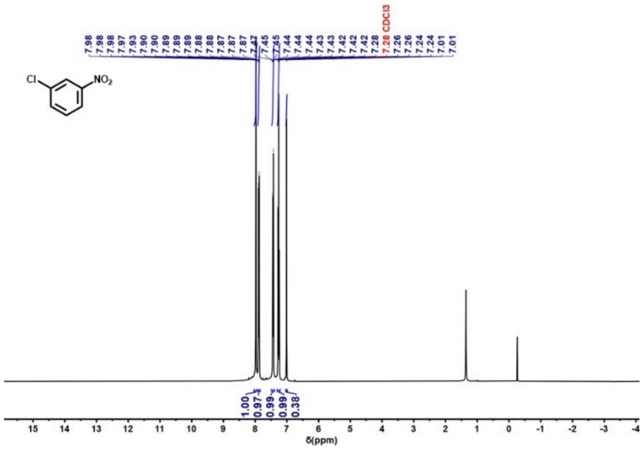

Fig. S37. ^{1}H NMR of m-chloronitrobenzene. ^{1}H NMR (400 MHz, Chloroform-*d*) δ 7.98 (q, *J* = 1.9 Hz, 1H), 7.88 (ddt, *J* = 8.3, 2.3, 1.2 Hz, 1H), 7.43 (ddt, *J* = 8.0, 2.2, 1.2 Hz, 1H), 7.31 – 7.21 (m, 1H).

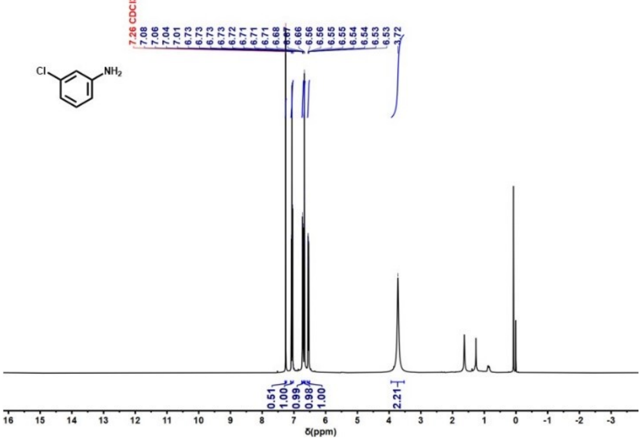

Fig. S38. ^{1}H NMR of m-Chloroaniline. ^{1}H NMR (400 MHz, Chloroform-*d*) δ 7.24 (s, 1H), 7.06 (t, *J* = 8.0 Hz, 1H), 6.72 (ddd, *J* = 7.9, 2.0, 0.9 Hz, 1H), 6.67 (t, *J* = 2.1 Hz, 1H), 6.54 (ddd, *J* = 8.0, 2.3, 0.9 Hz, 1H), 3.72 (s, 2H).

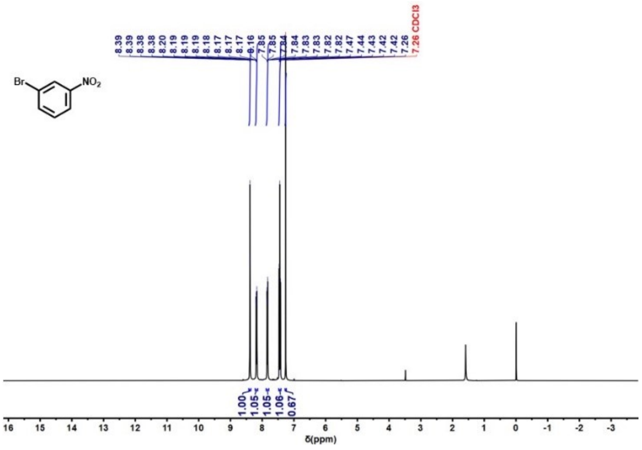

Fig. S39. ^{1}H NMR of M-bromonitrobenzene. ^{1}H NMR (400 MHz, Chloroform-*d*) δ 8.39 (t, *J* = 2.0 Hz, 1H), 8.18 (ddd, *J* = 8.3, 2.2, 1.0 Hz, 1H), 7.84 (ddd, *J* = 8.0, 1.9, 1.0 Hz, 1H), 7.44 (t, *J* = 8.1 Hz, 1H), 7.26 (s, 1H).

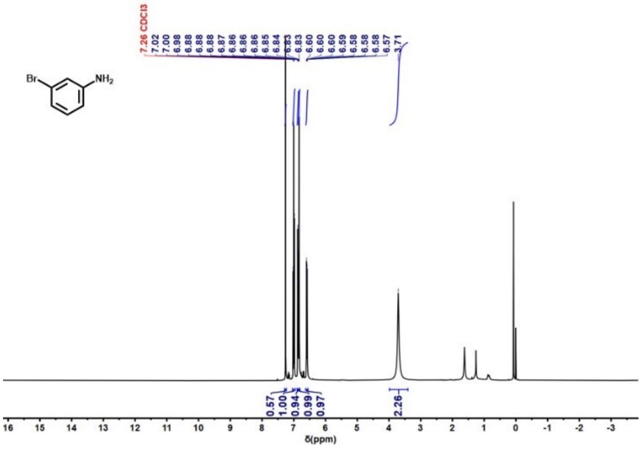

Fig. S40. ^{1}H NMR of M-bromoaniline. ^{1}H NMR (400 MHz, Chloroform-*d*) δ 7.00 (t, *J* = 7.9 Hz, 1H), 6.87 (ddd, *J* = 7.8, 1.9, 0.9 Hz, 1H), 6.83 (t, *J* = 2.1 Hz, 1H), 6.59 (ddd, *J* = 7.9, 2.3, 0.9 Hz, 1H), 3.71 (s, 2H).

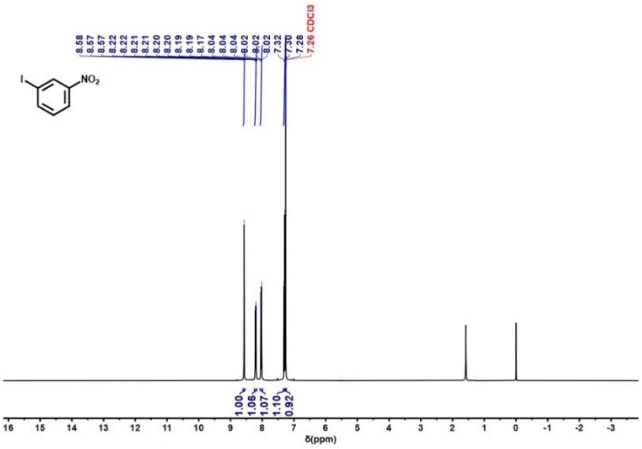

Fig. S41. ^{1}H NMR of M-iodonitrobenzene. ^{1}H NMR (400 MHz, Chloroform-*d*) δ 8.57 (t, *J* = 1.9 Hz, 1H), 8.21 (ddd, *J* = 8.3, 2.3, 1.0 Hz, 1H), 8.03 (dt, *J* = 7.8, 1.3 Hz, 1H), 7.30 (t, *J* = 8.1 Hz, 1H).

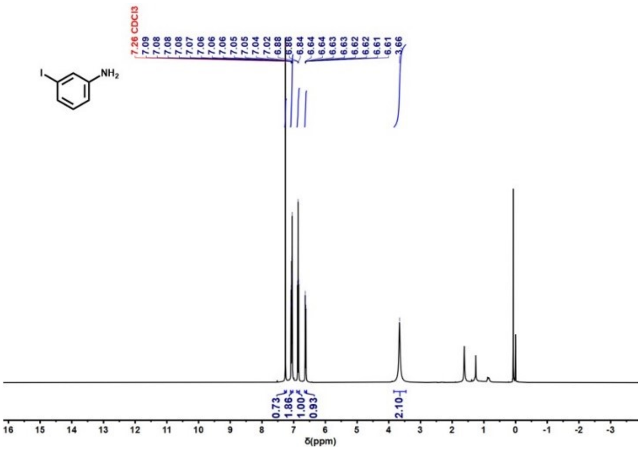

Fig. S42. ^{1}H NMR of M-iodoaniline. ^{1}H NMR (400 MHz, Chloroform-*d*) δ 7.28 (s, 1H), 7.10-7.02 (m, 2H), 6.86 (t, *J* = 7.9 Hz, 1H), 6.62 (ddd, *J* = 8.0, 2.2, 0.9 Hz, 1H), 3.66 (s, 2H).

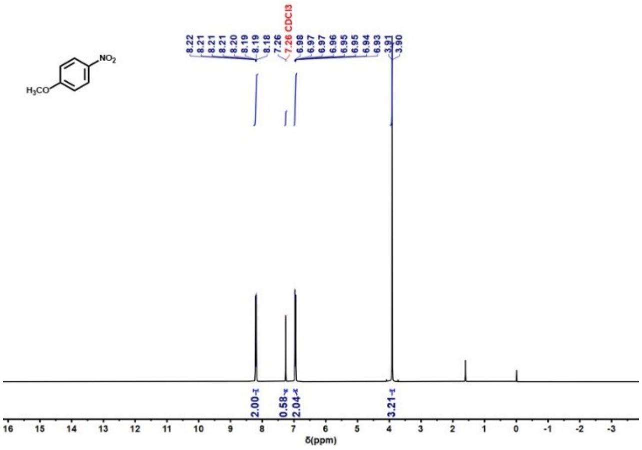

Fig. S43. ^{1}H NMR of P-nitroanisole. ^{1}H NMR (400 MHz, Chloroform-*d*) δ 8.28-8.13 (m, 2H), 7.26 (s, 1H), 7.01 – 6.91 (m, 2H), 3.91 (d, *J* = 1.2 Hz, 3H).

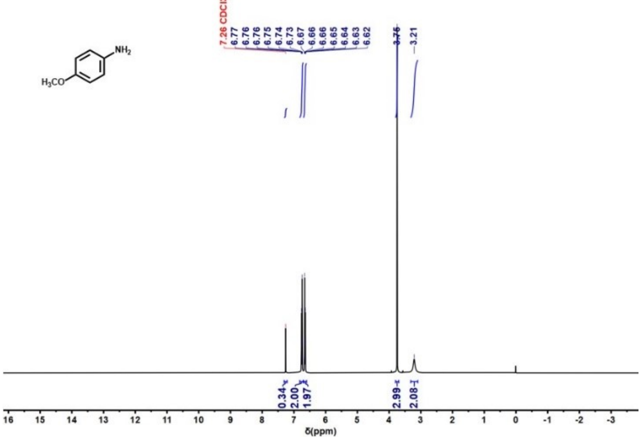

Fig. S44. ^{1}H NMR of p-Anisidine. ^{1}H NMR (400 MHz, Chloroform-*d*) δ 6.82-6.71 (m, 2H), 6.70 – 6.60 (m, 2H), 3.75 (s, 3H), 3.21 (s, 2H).

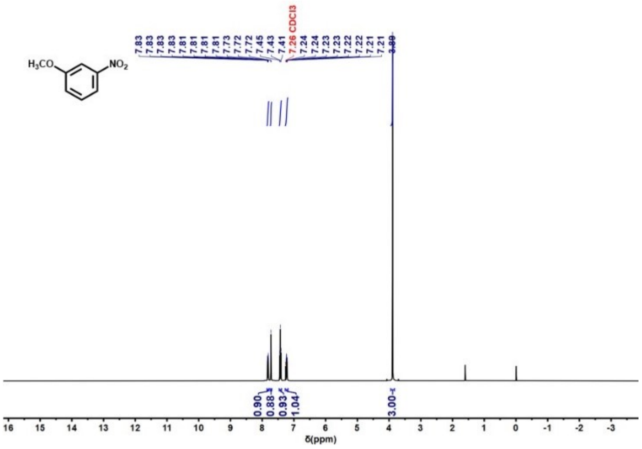

Fig. S45. ^{1}H NMR of M-nitroanisole. ^{1}H NMR (400 MHz, Chloroform-d) δ 7.82 (ddd, J = 8.2, 2.1, 0.9 Hz, 1H), 7.72 (t, J = 2.3 Hz, 1H), 7.43 (t, J = 8.2 Hz, 1H), 7.22 (ddd, J = 8.3, 2.6, 0.9 Hz, 1H), 3.89 (s, 3H).

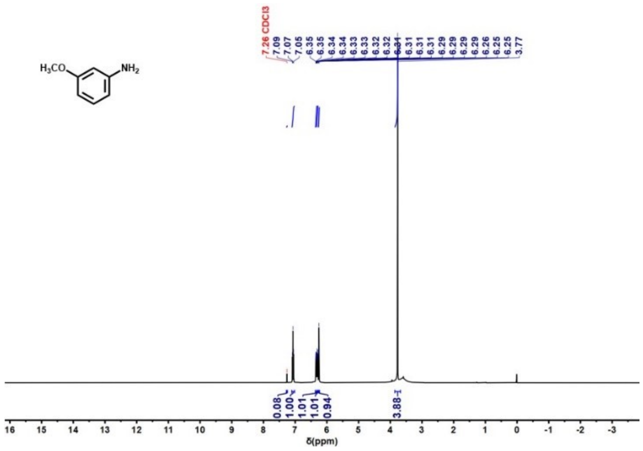

Fig. S46. ^{1}H NMR of M-Methoxyaniline. ^{1}H NMR (400 MHz, Chloroform-*d*) δ 7.07 (t, *J* = 8.0 Hz, 1H), 6.34 (ddd, *J* = 8.2, 2.4, 0.9 Hz, 1H), 6.30 (ddd, *J* = 7.8, 2.2, 0.9 Hz, 1H), 6.25 (t, *J* = 2.3 Hz, 1H), 3.77 (s, 4H).

Table S1. Details of the XPS analysis.

catalysts	Peak B.E. (FWHM) /eV						
	Mo^{2+}		Mo^{4+}		Mo^{6+}		S2p
	$3d_{5/2}$	$3d_{3/2}$	$3d_{5/2}$	$3d_{3/2}$	$3d_{5/2}$	$3d_{3/2}$	
Mo_2C	228.3<br>(0.8)	231.4<br>(0.9)	228.9<br>(1.0)	232.0<br>(1.0)	232.7<br>(2.2)	235.3<br>(2.2)	/
$Mo_2C@MoS_2$	228.2<br>(1.0)	231.3<br>(1.0)	228.8<br>(0.9)	231.9<br>(1.0)	/	/	226.0<br>(2.2)
MoS_2	/	/	228.7<br>(0.9)	231.8<br>(1.0)	/	/	226.0<br>(2.2)

Table S2. ECH performance of nitroarenes to anilines over the recently reported electrocatalysts.

catalysts	substrates	*E*	F.E.	sel.	yield	electrolytes	Ref.
$Mo_2C@MoS_2$	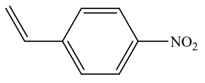	-0.45 V vs. RHE	85%	99%	78%	0.1 M $LiClO_4$	**This work**
Mo_2C	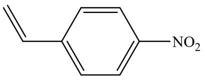	-0.45 V vs. RHE	23%	87%	13%	0.1 M $LiClO_4$	**This work**
MoS_2	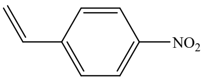	-0.45 V vs. RHE	8%	57%	4%	0.1 M $LiClO_4$	**This work**
Pd-Mo	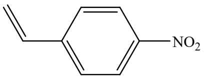	-0.25V vs RHE	84%	93%	63%	0.1 M $LiClO_4$	1
Co_3S_{4-x}	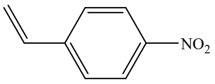	-1.0 V vs Hg/HgO	/	96%	86%	1.0 M KOH	2
CoP	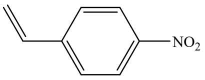	-1.2 V vs Ag/AgCl	/	92%	86%	1.0 M KOH	3
$CuCo_2O_4$/NF	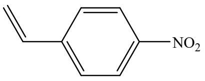	-1.0 V vs. SHE	/	/	93%	1.0 M KOH	4
Cu_3Pt	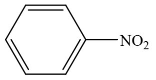	0.35V vs RHE	/	~99%	/	1.0 M KOH	5
Au@PtNi	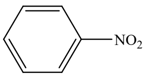	-0.2V vs RHE	/	82%	/	10 mg L^{-1} NB solution	6
Ag/LIG	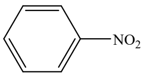	-0.88V vs RHE	48%	94%	/	0.1 M PB	7
PA-CF	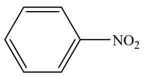	-0.53V vs RHE	/	~99%	/	0.3 M $HClO_4$/ethanol	8
NOMC	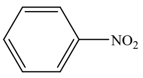	-0.75V vs Fe/Fe^+	/	87%	/	0.3 M $HClO_4$/ethanol	9
Co_9S_8/Ni_3S_2-	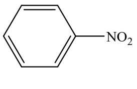	-0.121V	95%	96%	/	1.0 M KOH	10

NF		vs RHE					
Cu/Ti-3	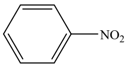	-0.9 V vs Ag/AgCl	/	97%	/	10 mM Na_2SO_4	11

Table S3. Experimentally observed Raman peaks and assignments.

Raman shift (cm^{-1})			Assignment
**Mo_2C**	**$Mo_2C@MoS_2$**	**MoS_2**	
1100	/	1100	C-N band stretching vibration
1150	1150	1150	CCH in-plane bend
1200	/	1200	N-O band stretching vibration
1400	1400	1400	N=O band stretching vibration
1450	1450	1450	ring stretch

Table S4. Details of the quasi *in situ* EIS analysis.

time	Mo_2C		$Mo_2C@MoS_2$		MoS_2	
	**equivalent circuits**	R_s	**equivalent circuits**	R_s	**equivalent circuits**	R_s
**0 min**	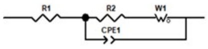	4.2	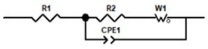	4.0	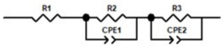	3.8
**10 min**	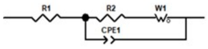	4.2	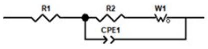	3.8	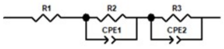	3.9
**20 min**	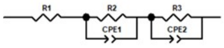	3.7	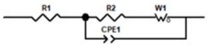	4.8	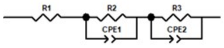	3.9
**30 min**	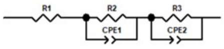	3.7	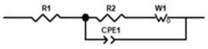	3.7	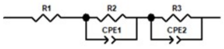	3.9
**40 min**	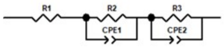	3.8	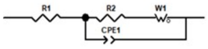	3.5	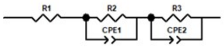	3.9
**50 min**	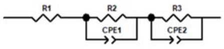	3.8	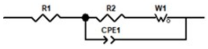	3.8	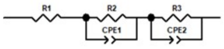	3.9
**60 min**	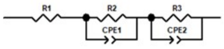	3.9	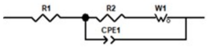	3.84	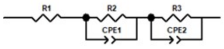	3.9

References

1. W. Zhang, W. Zhang, J. Tan, D. Pan, Y. Tang and Q. Gao, Alloying promotion of Pd-based metallenes in electrocatalytic hydrogenation of functionalized nitroarenes, *Journal of Materials Chemistry A*, 2023, **11**, 7505-7512.
2. Y. Zhao, C. Liu, C. Wang, X. Chong and B. Zhang, Sulfur vacancy-promoted highly selective electrosynthesis of functionalized aminoarenes via transfer hydrogenation of nitroarenes with H_2O over a Co_3S_{4-x} nanosheet cathode, *CCS Chemistry*, 2021, **3**, 507-515.
3. X. Chong, C. Liu, Y. Huang, C. Huang and B. Zhang, Potential-tuned selective electrosynthesis of azoxy-, azo-and amino-aromatics over a CoP nanosheet cathode, *National science review*, 2020, **7**, 285-295.
4. S. Wu, X. Huang, H. Zhang, Z. Wei and M. Wang, Efficient electrochemical hydrogenation of nitroaromatics into arylamines on a $CuCo_2O_4$ spinel cathode in an alkaline electrolyte, *ACS Catalysis*, 2021, **12**, 58-65.
5. M. Jin, Y. Liu, X. Zhang, J. Wang, S. Zhang, G. Wang, Y. Zhang, H. Yin, H. Zhang and H. Zhao, Selective electrocatalytic hydrogenation of nitrobenzene over copper-platinum alloying catalysts: Experimental and theoretical studies, *Applied Catalysis B: Environmental,* 2021, **298**, 120545.
6. J. Ma, Z. Wang, T. Majima and G. Zhao, Role of Ni in PtNi Alloy for Modulating the Proton-Electron Transfer of Electrocatalytic Hydrogenation Revealed by the In Situ Raman–Rotating Disk Electrode Method, *ACS Catalysis*, 2022, **12**, 14062-14071.
7. W. Li, J.-W. Zhao, C. Yan, B. Dong, Y. Zhang, W. Li, J. Zai, G.-R. Li and X. Qian, Asymmetric activation of the nitro group over a Ag/Graphene heterointerface to boost highly selective electrocatalytic reduction of nitrobenzene, *ACS Applied Materials Interfaces*, 2022, **14**, 25478-25489.
8. Y. Gao, Q. Xue, J. Li, M. Zhang, Y. Ma and Y. Qu, Phytate Coordination-Enhanced Electrocatalytic Activity of Copper for Nitroarene Hydrogenation

through Concerted Proton-Coupled Electron Transfer, *ACS Applied Materials & Interfaces*, 2022, **14**, 14202-14209.

9. N. Daems, F. Risplendi, K. Baert, A. Hubin, I. F. Vankelecom, G. Cicero and P. P. Pescarmona, Doped ordered mesoporous carbons as novel, selective electrocatalysts for the reduction of nitrobenzene to aniline, *Journal of Materials Chemistry A*, 2018, **6**, 13397-13411.

10. X. Wang, L. Li, M. Shi, Y. Wang, G. Xu, K. Yuan, P. Zhu, M. Ding and Y. Chen, Understanding the electrocatalytic mechanism of self-template formation of hierarchical Co_9S_8/Ni_3S_2 heterojunctions for highly selective electroreduction of nitrobenzene, *Chemical Science*, 2022, **13**, 11639-11647.

11. Y. Chen, L. Xiong, W. Wang, X. Zhang and H. Yu, Efficient and selective electro-reduction of nitrobenzene by the nano-structured Cu catalyst prepared by an electrodeposited method via tuning applied voltage, *Frontiers of Environmental Science & Engineering*, 2015, **9**, 897-904.
